# Failure mode and effect analysis in the indwelling urinary catheterization process*

**DOI:** 10.1590/1518-8345.7589.4795

**Published:** 2026-02-02

**Authors:** Caroline Zottele Piasentin Giacomini, Ana Elisa Bauer de Camargo Silva, Janete de Souza Urbanetto, Paulo Sousa, Tania Solange Bosi de Souza Magnago

**Affiliations:** 1 Universidade Federal de Santa Maria, Santa Maria, RS, Brazil; 2 Universidade Federal de Goiás, Faculdade de Enfermagem, Goiânia, GO, Brazil; 3 Pontifícia Universidade Católica do Rio Grande do Sul, Escola de Ciências da Saúde e da Vida, Porto Alegre, RS, Brazil; 4 Universidade NOVA de Lisboa, Escola Nacional de Saúde Pública, Lisboa, LX, Portugal

**Keywords:** Patient Safety, Risk Management, Health Care Quality, Healthcare Failure Mode and Effect Analysis, Nursing Care, Urinary Catheters

## Abstract

to analyze potential risks in the adult indwelling urinary catheterization process.

exploratory, descriptive and evaluative research in a teaching hospital. Working group with ten health care providers: eight nurses, one physician and one nursing technician. Activity flowchart and description built through process modeling. Potential risk analysis based on the Failure Mode and Effect Analysis method.

four sub-processes identified for the indwelling urinary catheterization process. Process review-based proactive health care risk analysis showed 55 potential failure modes, 92 potential failure causes, and 40 potential failure effects.

the method applied to review the indwelling urinary catheterization process in the hospital setting supported the proactive health care risk analysis; this issue should be further considered to contribute to the safety culture at the national level. Nurses are strategic in decision-making on health care risk management in said process, throughout catheter introduction, continuous use, and removal.

## Highlights

**(1)** Proactive risk analysis in a health care process. **(2)** Modeling of the indwelling urinary catheterization process. **(3)** Nurses are strategic in managing the indwelling urinary catheterization process.

## Introduction

Health care risk management is an essential aspect for patient safety in health care services, as it seeks to identify and address the risks to which patients, health care providers and health care organizations are exposed^(1)^. From this perspective, Failure Mode and Effect Analysis (FMEA) is a tool that can be used proactively to prevent safety incidents. It is adequate and effective for risk management of any nature^(1)^.

FMEA is applied transversally, considering concepts related to the failure chain, such as potential failure mode (PFM), potential failure cause (PFC) and potential failure effect (PFE). In addition, the verification can also consider the Severity of PFE, the probability of Occurrence of PFC and the probability of Detection/Prevention of PFM that can affect patients^(2)^.

Notable health care processes that require risk management include the Indwelling Urinary Catheter (IUC) process. This is a routine invasive intervention, which can cause complications related to Urinary Tract Infection (UTI) and others with damage to the lower urinary tract, such as pain, urethral trauma, false path, urethral fistula, prostatitis, epidymitis^(3)^. The procedure consists in introducing a sterile probe into the bladder through the urethra, with the main purpose of facilitating the drainage of urine, keeping the bladder catheterized with a probe connected to a sterile closed system for hours or days.

The urinary catheter (UC) is inserted, maintained and removed by nurses, while physicians are responsible for requesting the insertion and removal. It should be noted that nurses are responsible for the IUC process. UC insertion, handling and removal are activities constituting the nursing process, with care that requires technical complexity, scientific knowledge and the ability to make immediate decisions^(4-5)^.

Approximately 15% to 25% of hospitalized patients are exposed to the IUC process, and although the catheter insertion procedure requires sterile technique, the most frequent complication is catheter-associated UTI (CAUTI)^(3)^.

Scientific evidence indicates a satisfactory level of knowledge regarding care in the insertion of IUC and the need to review daily use^(6)^. However, deficiencies in relation to knowledge about the practices that are part of the procedure, especially in terms of indications for the use of IUC and care in its maintenance, are still noted.

The institution participating in the study has validated CAUTI prevention protocol and standard operating procedure (SOP) for the passage, maintenance and handling of IUC. However, incidents related to this care process continue to occur^(7)^. In this regard, in order to improve the care in relation to the use of IUC, it is necessary to identify the failures and gaps of this care practice from the indication to the removal of the catheter^(8)^. By evaluating an invasive process that can result in adverse events for patients, through a proactive tool, the objective is to identify the weakest points and propose improvements to prevent or eliminate potential failures, increasing the reliability, safety and quality of the IUC process.

Establishing a culture of open communication and learning from mistakes, focusing on non-punishment and the identification of risk factors, can provide reduced cases of these health care-related incidents^(9)^. This enables the implementation of specific interventions that promote change in the professionals’ habits and expectations about the need, safe use of IUC, and management of adverse events, such as CAUTI and urethral trauma^(8)^.

From this perspective, the objective was to analyze the potential risks in the IUC process in adults.

## Method

### Study design

This is an exploratory and descriptive study that evaluated the improvement of the quality of the IUC care process, following the recommendations of SQUIRE 2.0 for clarity and transparency in writing^(10)^.

### Study field, location and period

The study was conducted in a teaching hospital in the central area of Rio Grande do Sul state, Brazil. The emergency room, medical and surgical hospitalization, and intensive care units were the main health care settings for adult patients listed for the study. Data collection was held between August and December 2022.

### Participant population, selection criteria, and definition

Health care professionals who provided direct care to adult patients and were members of the Patient Safety Center and the Health Care Infection-Related Control Committee were invited to participate in the study, totaling 40 professionals. We applied inclusion criteria, such as working in the adult health care area, being involved in the listed care process and having worked at least six months or more in the institution. The exclusion criteria were being in a leave from professional activities at the time of data collection, sick leave, special leave or vacation.

The sample was non-probabilistic and by convenience. After the invitation and application of the selection criteria, the multidisciplinary working group (WG) was formed with ten health care professionals, who confirmed the availability to develop the method^(1)^. Of these, four care nurses from the adult care area, one infectologist and intensive care physician, one nursing technician from the adult intensive care unit, two nurses from the infection control service, one nurse from the quality sector and the hospital’s health care risk manager.

### Data collection and obtainment

The seven steps recommended by the FMEA method^(1)^ were applied. System analysis had three steps (planning and preparation; structure analysis, and function analysis). In the planning and preparation (step 1), the researcher made a schedule of deadlines and tasks with the WG, listing tools (brainstorming, flowchart and Ishikawa diagram), forms and support tables recommended by proactive risk analysis^(1)^. Structure analysis and function analysis (steps 2 and 3) were carried out by the WG, through the preparation of a process flowchart with the description of potential failures (Figure 1).

Failure analysis and risk mitigation occurred in three other steps (failure analysis, risk analysis and optimization). In establishing the failure chain (step 4), the WG used questions to identify the PFM, PFE and PFC. For risk analysis (step 5), the PFE Severity and PFC Occurrence indices were obtained. For the optimization (step 6), the WG assigned responsibilities and deadlines for implementing the actions (Figure 1).

Finally, in the risk communication (step 7), the WG defined the content of the documentation of the results, conclusions and analysis. Finally, the Risk Priority Number (RPN) was used by the WG to prioritize high-risk failures and identify the necessary actions to minimize their impacts (Figure 1).

**Figure 1–:** Steps conducted by the FMEA Method*. Santa Maria, RS, Brazil, 2023

FMEA* method						
a. System analysis			**b. Failure analysis and risk mitigation**			**c. Risk communication**
*Step 1. Planning and preparation*	***Step 2. Structure analysis***	***Step 3. Function analysis***	***Step 4. Failure analysis***	***Step 5. Risk analysis***	***Step 6. Optimization***	***Step 7. Documentation of results and conclusions and analysis***
*Project Plan* (5T): InTent, Timing, Team, Tasks, Tools	Structure and Function Tree or equivalent: *process flowchart*	*Failure chain establishment*	*Assignment of Prevention Controls for Potential Failure Modes and Causes*	*Assignment of responsibilities* and *deadlines* for implementation of actions	*Definition* of the *documentation* content
*Step 1:* InTent: object of this research *Timing* : August to December 2022 *Team* : multidisciplinary working group *Tasks* : meetings *Tools* : flowchart, *brainstorming* and Ishikawa diagram	*Step 2:* Construction, and evaluation, of the flowchart and description of the potential failures	*Step 3:* Identify the Potential Failure Mode (What can go wrong?) *Step 4:* Identify the Potential Failure Effect (What happens? What would be the consequences of the failure?) *Step 5:* Identify the Potential Failure Cause (Why does the failure happen?)	*Step 4.1:* Obtain Severity Indices (Severity of Potential Failure Effect) *Step 5.1:* Obtain Occurrence Indices (Probability of Occurrence of Potential Failure Cause) *Step 6:* Determine current existing and/or planned control measures and failure scores *Step 6.1:* Obtain Detection Indices (Probability of Failure Detection) *Step 7:* Calculate the RPN ^†^ (Severity of effect x Occurrence of Potential failure cause x Detection of failure)	*Step 8:* Use the RPN ^†^ to prioritize high-risk failures *Step 9:* Identify the actions needed to reduce risks

Source: Adapted^(2)^; *FMEA = Failure Mode and Effect Analysis; ^†^RPN= Risk Priority Number

### Data organization, analysis and presentation

Process modeling used the free software Bizagi Modeler ^®^ (version 3.4.0.062/2019) to design, diagram, document and publish the processes, using the Business Process Modeling Notation (BPMN).

All data from the meetings were transcribed to an electronic database of the Microsoft Excel^®^ program. After the WG validated the typed data by double checking the database, we generated the analyses, as described in Figure 1, and the reports.

### Ethical aspects

The research was registered and approved by the Research Ethics Committee of the Federal University of Santa Maria under number 58032622.6.0000.5346.

## Results

The Working group had ten health care providers: eight nurses, one physician and one nursing technician. There was a predominance of female health professionals (n=9; 90%), with employment association according to the Brazilian Consolidation of Labor Laws (CLT) (n=7; 70%), who work in the afternoon (n=4; 40%) and have a Master’s degree in health sciences (n=6; 90%).

For application of the FMEA method, the WG held 20 meetings, lasting two hours each, totaling 40 hours. The discussion of sub-process 1 required three meetings; sub-processes 2 and 4, four meetings; and sub-process 3, nine meetings.

**Figure 2-:**
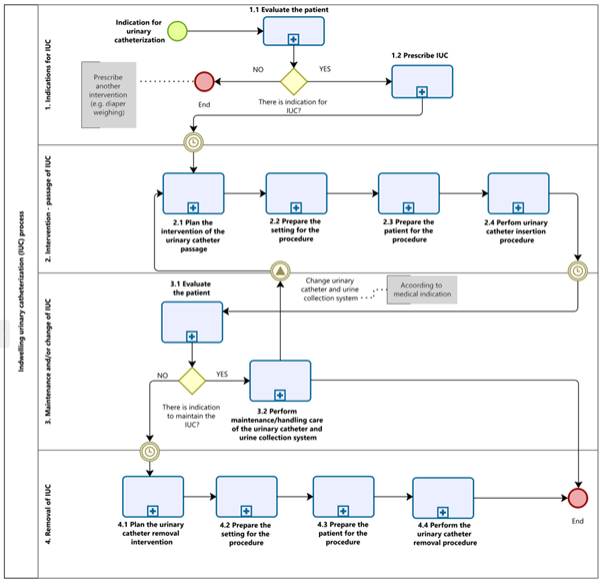
Indwelling urinary catheterization process. Santa Maria, RS, Brazil, 2023

Through information gathered in the brainstorming sessions, the WG mapped the IUC process. After that, four key steps were described and named as sub-processes: 1) Indications for IUC; 2) Intervention – passage of IUC; 3) Maintenance and/or exchange of IUC; and 4) Removal of IUC (Figure 2). The developments of these sub-processes were described as activities and tasks.

Four sub-processes, 12 activities and 70 tasks were mapped. We obtained three professional categories with assignments, competencies and responsibilities. Physicians begin subprocesses 1) Indications for IUC and 4) Removal of IUC, and nurses are involved in subprocesses 2) Intervention – passage of IUC and 3) Maintenance and/or change of IUC with the help of nursing technicians.

As for the potential failure modes, causes and effects in the IUC process, we identified 55 potential failure modes, 92 potential failure causes and 40 potential failure effects.

In subprocess 1) Indications for IUC, we found 6 (10%) potential failure modes, 14 (15%) potential failure causes, and 4 (10%) potential failure effects. We note the PFM *unaware of the indications for* IUC with high Priority of Action (PA). Presenting the PFC *absence of insertion indication in the urinary tract infection (CAUTI) prevention protocol* with *unnecessary catheterization* as PFE.

Subprocess 2) Intervention – passage of IUC showed the largest number of activities and tasks, consequently presenting the largest number of modes, causes and effects found. Namely: 43 (78%) potential failure modes, 71 (78%) potential failure causes, and 26 (66%) potential failure effects.

In this subprocess, the WG addressed the tasks necessary for the nursing procedure to achieve the indication proposed. The PFM *not sanitizing the urinary meatus* showed high PA, indicating as potential failure causes *unaware of the SOP for IUC insertion* and *not caring about the hygiene of the urinary meatus (knowing the technique and not performing it)* as main contributors to the PFE *risk of CAUTI; break of aseptic technique*.

The activity related to Hand Hygiene (HH) permeated sub-process 2, obtaining high PA in two potential failures modes related to *inadequate HH - either by inadequate technique and/or time (s); by the loss of opportunities for HH* and *not sanitizing the hands*, having as potential failure causes the *lack of access to infrastructure (sinks away from the beds), work overload; multiple tasks*, *disinterest in knowing the hand hygiene protocol available on the hospital intranet – not caring about HH*, *lack of access to infrastructure (lack of dispensers of alcoholic solution at the bedside)* and *disinterest in sanitizing the hands* being contributors to the potential failure effects related to the act of *not sanitizing the hands - increasing the risk of Health Care-Associated Infections (HAIs)* and *low adherence to hand hygiene - increasing the risk of HAIs.*

Another PFM reported with high PA was *not having an auxiliary support table for the IUC insertion procedure*, having as PFC the *lack of an auxiliary table or cart* and contributing to the PFE *risk of contamination of materials, because they are in an inadequate place (for example, the patient’s bed)*.

In subprocess 3) Maintenance and/or change of IUC, three (5%) potential failure modes, three (3%) potential failure causes, and three (7%) potential failure effects were found. Notably, the potential failure modes *fixing the foley probe with inadequate product (for example: micropore or non-sterile film)* and *not fixing the foley probe* with high PA, respectively. Having as main potential failure causes the *lack of fixator – product suitable for the routine* and *not fixing the foley probe due to forgetfulness* causing the potential failure effects *skin lesion by device* or *bleeding or scarification of the urethra – risk of traction of the foley probe – risk of CAUTI by traction of the collector system.*

Corroborating, it was shown that the PFM *not having assistance during the IUC procedure* permeated subprocesses 2 and 3, presenting as main potential failure causes the *lack of professionals to compose the nursing team,work overload; multiple tasks*, *nursing technician not assisting the nurse responsible for the passage of the IUC*, being the main contributors to the PFE *risk of CAUTI; break of aseptic technique*.

In subprocess 4) IUC removal, three (5%) potential failure modes, four (4%) potential failure causes, and seven (17%) potential failure effects were found. No PFM presented high PA, as the subprocess presented activities discussed by the WG in subprocess 2.

However, although the potential failure modes related to sub-process 4 have shown low PA, we report the PFM *not adequately deflating the foley probe cuff* having as PFC *material without quality* contributing to the potential failure effects related to *urethral trauma*, *not being able to remove the foley probe from the urinary meatus* or *cuff popping and pieces of material (latex) remaining in the patient’s bladder.*

The potential failure causes (n=92; 100%) were inserted in the Ishikawa diagram, being reclassified and categorized into causes related to the use of equipment (n=21; 23%), behavior/skill/attitude (n=18; 19%), education and training (n=17; 18%), social or teamwork (n=12; 13%), activity/task (n=11; 12%), patient (n=5; 6%), work environment (n=5; 6%) and communication (n=3; 3%).

## Discussion

The classification of patients in terms of presence (or not) and appropriate use of IUC can be based on guidelines^(3,11)^. In the period, it was found that the CAUTI prevention protocol of the researched institution did not include the main indications for insertion of the urinary catheter. Although the clinical evaluation for IUC is sovereign, the indications^(3)^ can guide good practices, highlighting the risks and benefits to assist physicians in decision-making.

Researchers^(12-13)^ point out that nurses and physicians are not familiar with the indications for IUC. A cohort study with 388 patients showed that one in four patients underwent placement of a bladder tube [24.7% (n=96); 95% Confidence Interval (95% CI: 20% to 29%)], and 36.5% (95% CI: 33% to 48%) of the cases did not present clinical criteria for such procedure^(12)^. Inappropriate use of IUC is more common in clinical than in surgical wards, and inappropriate use of the device occurred in 14 (10.4%) of 134 surgical patients, compared to 105 (32.4%) of 324 clinical patients, a difference of 22% (95% CI: 14.7-29.2; p<0.001)^(13)^.

Regarding unnecessary catheterization, studies^(14-15)^ with health care professionals aimed to perform interventions to reduce UC insertion and, consequently, infectious and non-infectious complications. They applied packages with measures to align the knowledge of physicians and nurses on indications for the use of catheters. Specific situations where urinary catheterization is unnecessary and educational actions that varied with time and methodology were included. A simple set of measures increased UC use with evidence-based indications and resulted in change in practice related to continued use of the device. CAUTI rates remained at low levels. However, it had no impact on non-infectious complications^(14-15)^.

When addressing the preparation of the setting for the IUC insertion procedure, an evaluation of the bed of the patient that will be submitted to the procedure should be performed. It is recommended that the bed assessment include the patient’s bed (pillow, cushions, sheets), available hospital medical materials such as side table, spotlight and screens to ensure patient privacy.

As for patient preparation for the procedure, regarding urinary meatus hygiene, there is no consensus in the literature on the choice of hygiene products, and the guidelines differ in their recommendations. The American guidelines^(3)^ provide no recommendation, stating that the problem remains unsolved and presents a research gap on the use of antiseptic solutions. The English guidelines^(11)^ recommend the use of saline solution for urethral meatus hygiene before catheter insertion.

HH permeated sub-process 2. In the hospital setting, HH is essential to avoid damage related to health care. It requires greater commitment on the part of everyone involved. In this regard, manager interventions to comply with infrastructure and inputs (sinks, faucets, dispensers, paper towels) are important to ensure the necessary conditions for health care professionals who provide direct patient care^(16)^.

Safety and quality of care in the nursing process related to IUC are not only related to professional qualification and prevention measures. There are other factors that can affect this health care process, such as resources, sufficient and quality materials, as well as adequate structure for execution of the procedure.

When addressing the insertion procedure, the WG noted the competencies of nurses and the duties of nursing technicians in situations requiring support for IUC insertion procedure. In this sense, it is necessary that nurses have scientific knowledge and technical skill, seeking a balance between patient safety and cost-benefit ratio^(17-18)^.

Regarding clinical practice, the guidelines of the European Association of Urology Nurses (EAUN) presents evidence-based guidelines to support a uniform IUC insertion practice in adults^(19-20)^. A Swedish study evaluated the impact of EAUN guidelines on aseptic technique during catheterization in two hospitals. The compliance of the procedure with aseptic technique was associated with the surgery and cardiology teams (Odds ratio (OR): 2.35; 95% CI: 1.69 - 3.27), the use of sterile “kit” for catheterization (OR: 2.06; 95% CI: 1.42 - 2.97), the use of sterile fields for the insertion area (OR: 1.91; 95% CI: 1.24 - 2.96) and the use of the term “sterile technique” for UC insertion (OR: 1.64; 95% CI: 1.11 - 2.43). The result showed that 55 to 74% of nurses practiced one or more precautions that ensured the aseptic technique of UC insertion, demonstrating a gap between the EAUN guidelines and actual performance. They mentioned improvement strategies related to monitoring the sterile technique during catheter insertion, to provide reduced risk of contamination and the implementation of a continuous UC insertion protocol with the presence of two people with a view to reducing CAUTI^(21)^.

In this regard, not having assistance from the nursing technician during the IUC insertion procedure highlights the need for cooperation during the insertion procedure. Corroborating this finding, the implementation of the insertion protocol with two professionals indicated that this, added to other strategies, such as the application of a task checklist, decreased the risk of CAUTI of the patients studied^(22)^.

In Brazil, the normative opinion for the work of the nursing team in bladder probing establishes guidelines aimed at the effective safety of patients submitted to the procedure^(4)^. It describes that the passage of the UC is private to the nurse and presents actions that must be conducted by the nursing team during the execution of the procedure.

However, there is a need for legislation that reports the mandatory presence of the nursing technician, with regard to responsibilities related to the preparation of the material and the setting, the positioning of the patient, the opening of the sterile material, the destination of the materials used and the waste generated and, when necessary, the submission to the laboratory of the material collected for examinations. These responsibilities may contribute to reducing the occurrence of incidents, supporting patient safety, with technical-scientific rigor and teamwork.

As for IUC maintenance, catheter fixation is a practice recognized as an important and essential aspect for device maintenance. It consists in using a device to attach a urinary foley catheter to the patient’s inner thigh (female) or lower abdomen (male). Improperly fixing it can lead to complications such as inadvertent removal, skin lesions, urethral trauma or erosion^(23)^.

A Canadian study found an overall prevalence of 18% (8/44) of catheter fixation. Of these, seven were fixed correctly, and the main method of fixation was a commercial adhesive (6/8; 75%)^(24)^. The skin lesion by device is related to the prolonged presence of the catheter and/or the lack of catheter fixation; it can cause discomfort, irritation of the skin and urethral epithelium, erythema at the edge of the meatus, including total loss of tissue thickness, and complete penile cleavage, penile disfigurement and sexual and urinary dysfunction^(25)^.

The guidelines^(3,19,25)^ recommend that if a permanent catheter cannot be avoided, it should be fixed to the patient’s body to prevent catheter pull and pressure on the urethral meatus. The recommendation is based on a low level of evidence. Therefore, prospective studies are needed to establish evidence-based guidelines. Care related to fixation of the urinary probe is the responsibility of the nursing team and should include actions to prevent injuries. In this context, making the team aware of the importance of actions to prevent pressure injuries associated with the invasive device is the first step.

After achieving its purpose, the UC should be removed. UC trapping may occur due to defective insufflation channel, malfunction, or fluid crystallization within the cuff. The causes of retained foley catheters and the method for dealing with each incident may vary^(26)^.

By analyzing the continuous UC process, it is possible to perceive a dichotomy between practice, training and scientific evidence. In this sense, it is observed that the consideration of aspects related to innovation and technology in publications associated with UC process risk management is scarce, as well as on other nursing procedures performed routinely^(27)^.

In contrast, initiatives to improve clinical practice and reduce incidents with adverse events have been studied. These initiatives mainly include the prevention of modifiable risk factors for CAUTI and urethral trauma^(7,28-30)^. Quality improvement programs to reduce the use of IUC using evidence-based intervention packages, focusing on avoiding unnecessary use, as well as promoting adequate catheter insertion, technique and maintenance, have been described in the literature^(31-32)^.

The situations and reasons for omission of care, which is when “the professional cannot perform the right action either in the planning phase or in the execution phase”^(33)^, permeated the risk analysis of the IUC process. These findings are in line with the literature that notes that the most frequent reasons for the omission of nursing care are related to human resources (workforce shortage), material resources and unexpected increase in patients^(34-35)^.

In this regard, the WG reported the normalization of deviance, a term used for a phenomenon that happens when people in an organization become so insensitive that deviant practices no longer seem wrong. Insensitivity occurs daily and is often perpetuated in teams over the years, without due importance until critical factors align towards the incident/damage^(36)^. Given the above, not caring about the hygiene of the urinary meatus (knowing the technique and not performing it) is an example of deviance from evidence-based practice and violation of infection control.

It should be noted that health care providers do not make decisions with the intention of increasing health care risks and causing harm to patients. However, deviances occur due to the break of process barriers or drivers such as time, cost, work overload and peer pressure^(36)^. In this context, evaluating the findings of this research from the perspective of these concepts can help minimize or avoid the normalization of deviance and omission of care. This can occur through actions instituted with transparency and performance improvement tools, so deviance change and prevention start at the front line, but are supported by managers.

As for the lack of adherence to good practices, it is important to observe and study the health care scenarios, processes, interpersonal relationships, in order to capture the reasons, considering the complexity of the decision-making process and the different perceptions among health care providers.

IUC is a multiprofessional issue, with shared responsibilities that oscillate between professional categories. When intervening is necessary to provide improvements, it is important that measures to raise awareness and communicate new routines and standards are directed to the entire health care team, especially physicians and nurses.

This study is expected to provide inputs for rethinking the problems and failures related to health care processes beyond nursing, with a view to minimizing risks in care and preventing incidents to patient safety. Thus, the leadership’s commitment to the use of IUC, in a restricted and rational manner, needs to be visible beyond the multidisciplinary group, considering the patient and companion to pave the way for improvement.

As for the limitations of this study, the scope of the FMEA carried out by the WG was limited to the IUC process in adults, addressing the four sub-processes presented. Sub-processes related to the transition of care, transfer and transport of patients with IUC, with continuous irrigation or bladder lavage, were not included in the scope. Groups of patients in specific age groups (older adults, pregnant women, newborns, babies and children) were not addressed, in addition to patient and family engagement.

Due to the WG not knowing the tool, the difficulty faced was its use. The application of the method demanded attention due to the complexity of the concepts and the chains of failures. However, after the incorporation and adaptation period, with the resumption of the method, whenever necessary, the FMEA enabled a collective discussion with reflective amplitude on the daily health care practice. Since the results related to the chain of failures are inherent to a teaching hospital, it is suggested the extension of the application of this tool to other institutions.

## Conclusion

The proactive health care risk analysis in the hospital setting supported the application of the tool in a health care process. This issue requires further consideration to contribute to the safety culture at the national level.

It enabled the WG to review the IUC process, increasing the perception of risks related to patient safety and discussing actions to make it safer and better. We mapped 4 sub-processes, 12 activities and 70 tasks that showed the correlations between the sub-processes/activities, health care professionals, and pieces of information. The proactive health care risk analysis of the IUC process in the hospital settings found 55 PFMs, 92 PFCs and 40 PFEs.

Sub-process 2 presented in its failure chains the greatest risks that need to be proactively managed. Nurses are strategic in decision-making on health care risk management in said process, throughout catheter introduction, continuous use, and removal.

The main recommendations suggested by the WG addressed the potential failure modes with high PA and were compiled in a list to provide improved outcomes related to the object of the study. They included initiatives related to the implementation of multidisciplinary discussions to avoid unnecessary use of IUC; review of the CAUTI prevention protocol and the IUC insertion SOP; institutionalization of the insertion checklist and application of a package for maintenance and handling of IUC, in conjunction with a training program with the involvement of academia. Finally, it recommended a survey of the needed improvements related to hospital infrastructure, the process of procuring hospital medical articles, and the sizing of nursing teams.

## References

[1] Stamatis DH (2015). The ASQ pocket guide to Failure Mode and Effect Analysis (FMEA).

[2] Automotive Industry Action Group, Verband der Automobilindustrie (2019). Failure Mode and Effect Analysis: Guide FMEA.

[3] Gould CV, Umscheid CA, Agarwal RK, Kuntz G, Pegues DA (2019). Guideline for prevention of catheter-associated urinary tract infections (2009).

[4] Conselho Federal de Enfermagem (BR) (2013). Parecer normativo para atuação da equipe de enfermagem em sondagem vesical.

[5] Miranda MEQ, Rosa MR, Novelli e Castro MC, Fontes CMB, Bocchi SCM (2023). Nursing protocols to reduce urinary tract infection caused by indwelling catheters: an integrative review. Rev Bras Enferm.

[6] Shadle HN, Sabol V, Smith A, Stafford H, Thompson JA, Bowers M (2021). A bundle-based approach to prevent catheter-associated urinary tract infections in the intensive care unit. Crit Care Nurse.

[7] Giacomini CZP (2023). Analysis of the mode and effect of failure in the bladder probing.

[8] Santos RCR, Almeida RGS, Costa RRO, Mazzo A (2020). Trauma by urethral catheters: self-confidence of the nurse in a simulated scenario. Renome.

[9] Silva BJR, Santos BDV, Andrade CR, Macedo ER, Andrade HS (2021). Nursing actions that promove the security of the patient in the hospitalar scope. Res Soc Dev.

[10] Ogrinc G, Davies L, Goodman D, Batalden P, Davidoff F, Stevens D (2016). SQUIRE 2.0-Standards for Quality Improvement Reporting Excellence-Revised Publication Guidelines from a Detailed Consensus Process. BMJ Qual Saf.

[11] Loveday H, Wilson JA, Pratt RJ, Golsorkhi M, Tingle A, Bak A (2014). Epic3: national evidence-based guidelines for preventing healthcare-associated infections in NHS hospitals in England. J Hosp Infect.

[12] Ghauri SK, Javaeed A, Abbasi T, Khan AS, Mustafa KJ (2019). Knowledge and attitude of health workers regarding catheter-associated urinary tract infection in tertiary care hospitals, Pakistan. J Pak Med Assoc.

[13] Laan BJ, Vos MC, Maaskant JM, Henegouwen MIB, Geerlings SE (2020). Prevalence and risk factors of inappropriate use of intravenous and urinary catheters in surgical and medical patients. J Hosp Infect.

[14] Blondal K, Ingadottir B, Einarsdottir H, Bergs D, Steingrimsdottir I, Steindorsdottir S (2016). The effect of a short educational intervention on the use of urinary catheters: a prospective cohort study. Int J Qual Health Care.

[15] Schweiger A, Kuster SP, Maag J, Züllig S, Bertschy S, Bortolin E (2020). Impact of an evidence-based intervention on urinary catheter utilization, associated process indicators, and infectious and non-infectious outcomes. J Hosp Infect.

[16] Magnago TSBS, Dal Ongaro J, Greco PBT, Lanes TC, Zottele C, Gonçalves NG (2019). Infrastructure for hand hygiene in a teaching hospital. Rev Gaúcha Enferm.

[17] Institute for Healthcare Improvement (2011). How-to Guide: prevent catheter-associated urinary tract infections.

[18] Ling R, Giles M, Searles A (2021). Administration of indwelling urinary catheters in four Australian Hospitals: cost-effectiveness analysis of a multifaceted nurse-led intervention. BMC Health Serv Res.

[19] Geng V, Cobussen-Boekhorst H, Farrell J, Gea-Sánchez M, Pearce I, Schwennesen T (2012). Catheterisation. Indwelling catheters in adults.

[20] Wagner KR, Bird ET, Coffield KS (2016). Urinary catheterization: a paradigm shift in difficult urinary catheterization. Curr Urol Rep.

[21] Kulbay A, Joelsson-Alm E, Tammelin A (2021). The impact of guidelines on sterility precautions during indwelling urethral catheterization at two acute-care hospitals in Sweden - a descriptive survey. BMC Nurs.

[22] Fletcher-Gutowski S, Cecil J (2019). Is 2-person urinary catheter insertion effective in reducing CAUTI?. Am J Infect Control.

[23] Shum A, Wong KS, Sankaran K, Goh ML (2017). Securement of the indwelling urinary catheter for adult patients: a best practice implementation. Int J Evid Based Healthc.

[24] Appah Y, Hunter KF, Moore KN (2016). Securement of the indwelling urinary catheter: a prevalence study. J Wound Ostomy Continence Nurs.

[25] Shenhar C, Mansvetov M, Baniel J, Golan S, Aharony S (2020). Catheter-associated meatal pressure injury in hospitalized males. Neurourol Urodyn.

[26] Patel AB, Osterberg EC, Satarasinghe PN, Wenzel JL, Akbani ST, Sahi SL (2023). Urethral Injuries: Diagnostic and Management Strategies for Critical Care and Trauma Clinicians. J Clin Med.

[27] Rodrigues AL, Torres FBG, Santos EAP, Cubas MR (2021). Process modeling: technological innovation to control the risk for perioperative positioning injury. Rev Bras Enferm.

[28] Alex J, Maneze D, Ramjan LM, Ferguson C, Montayre J, Salamonson Y (2022). Effectiveness of nurse-targeted education interventions on clinical outcomes for patients with indwelling urinary catheters: A systematic review. Nurse Educ Today.

[29] Gad MH, Abdelaziz HH (2021). Catheter-associated urinary tract infections in the adult patient group: a qualitative systematic review on the adopted preventative and interventional protocols from the literature. Cureus.

[30] Pajerski DM, Harlan MD, Ren D, Tuite PK (2022). A clinical nurse specialist-led initiative to reduce catheter-associated urinary tract infection rates using a best practice guideline. Clin Nurse Spec.

[31] Joseph JT, Roy SS, Shams N guest, Visintainer P, Wormser GP (2022). A collaborative approach intended to reduce the duration of short term urinary catheters in adult patients at a tertiary care medical center also significantly reduced the duration of hospitalization. Am J Infect Control.

[32] Niederhauser A, Züllig S, Marschall J, Schweiger A, John G, Kuster SP (2019). Change in staff perspectives on indwelling urinary catheter use after implementation of an intervention bundle in seven Swiss acute care hospitals: results of a before/after survey study. BMJ Open.

[33] World Health Organization (2011). Estrutura Concetual da Classificação Internacional sobre Segurança do Doente.

[34] Lima JC, Silva AEBC, Caliri MHL (2020). Omission of nursing care in hospitalization units. Rev. Latino-Am. Enfermagem.

[35] Mota EC, Oliveira AC (2019). Prevention of catheter-associated urinary tract infection: what is the gap in clinical practice?. Texto Contexto Enferm.

[36] Price MR, Williams TC (2018). When doing wrong feels so right: normalization of deviance. J Patient Saf.

