# Supplementary material for: Failure mode and effect analysis in the indwelling urinary catheterization process
Source: Rev Lat Am Enfermagem. 2026 Feb 2;34:e4795. doi: 10.1590/1518-8345.7589.4795 (PMC12863658; doi:10.1590/1518-8345.7589.4795)
Supplement: Translation

# Translation: Análise do modo e efeito da falha no processo de cateterismo vesical de demora*

**DOI:** 10.1590/1518-8345.7589.4796

**Authors:** Caroline Zottele Piasentin Giacomini, Ana Elisa Bauer de Camargo Silva, Janete de Souza Urbanetto, Paulo Sousa, Tania Solange Bosi de Souza Magnago

**Affiliations:** 1 Universidade Federal de Santa Maria, Santa Maria, RS, Brasil; 2 Universidade Federal de Goiás, Faculdade de Enfermagem, Goiânia, GO, Brasil; 3 Pontifícia Universidade Católica do Rio Grande do Sul, Escola de Ciências da Saúde e da Vida, Porto Alegre, RS, Brasil; 4 Universidade NOVA de Lisboa, Escola Nacional de Saúde Pública, Lisboa, LX, Portugal

**Keywords:** Segurança do Paciente, Gestão de Riscos, Qualidade da Assistência à Saúde, Análise do Modo e do Efeito de Falhas na Assistência à Saúde, Cuidados de Enfermagem, Cateteres Urinários

## Abstract

analisar os riscos potenciais no processo de cateterismo vesical de demora em adultos.

pesquisa exploratória, descritiva e de avaliação realizada em um hospital universitário. O grupo de trabalho foi composto por dez profissionais de saúde, sendo oito enfermeiros, um médico e um técnico de enfermagem. A construção do fluxograma e descrição das atividades foi realizada por meio de modelagem do processo. A análise dos riscos potenciais com base no método *Failure Mode and Effect Analysis*.

foram identificados quatro subprocessos para o processo de cateterismo vesical de demora. A análise proativa de riscos assistenciais relacionados à revisão do processo evidenciou 55 modos potenciais de falha, 92 causas potenciais de falha e 40 efeitos potenciais de falha.

a aplicabilidade do método utilizado para a revisão do processo de cateterismo vesical de demora no ambiente hospitalar fortaleceu a análise proativa de riscos relacionados à saúde, temática que carece de maior visibilidade para contribuir com a cultura de segurança em âmbito nacional. Destacou a posição estratégica dos enfermeiros na tomada de decisão para gestão de riscos relacionados à prática assistencial do processo em destaque, desde a inserção e o uso contínuo até a remoção do dispositivo.

## Destaques

**(1)** Análise proativa de riscos realizada em um processo assistencial. **(2)** Modelagem do processo de cateterismo vesical de demora. **(3)** Enfermeiros são estratégicos para gerenciar o processo de cateterismo vesical de demora.

## Introdução

A gestão de riscos assistenciais de saúde é um aspecto essencial para a segurança do paciente nos serviços, pois busca identificar e tratar os riscos a que os pacientes, os profissionais e as organizações de saúde estão expostos^(1)^. Nessa perspectiva, a Análise de Modo e Efeitos de Falha (FMEA – do inglês *Failure Mode and Effect Analysis*) – é uma ferramenta que pode ser utilizada de forma proativa para prevenção de incidentes de segurança. Ela é adequada e eficaz para gestão de riscos de qualquer natureza^(1)^.

A aplicabilidade do FMEA acontece de forma transversal, considerando os conceitos relacionados à cadeia de falhas, como o modo potencial de causa (MPF), a causa potencial de falha (CPF) e o efeito potencial de falha (EPF). Além disso, também se pode verificar a Severidade do EPF, a probabilidade de Ocorrência da CPF e a probabilidade de Detecção/Prevenção do MPF que podem atingir o paciente^(2)^.

Entre os processos assistenciais que necessitam de gestão de riscos, destaca-se o processo de Cateterismo Vesical de Demora (CVD). Uma intervenção invasiva rotineira, capaz de causar complicações relacionadas à Infecção do Trato Urinário (ITU) e outras com dano ao trato urinário inferior, como dor, traumatismo uretral, falso trajeto, fístula uretral, prostatite, epidimite^(3)^. O procedimento consiste na inserção de uma sonda estéril até a bexiga, por meio da uretra, tendo como principal finalidade facilitar a drenagem da urina, mantendo a bexiga cateterizada com uma sonda ligada a um sistema fechado estéril durante horas ou dias.

O cateter vesical (CV) é inserido, mantido e removido por enfermeiros, enquanto os médicos são responsáveis por solicitar a inserção e remoção. Cabe destacar que o processo de CVD é prerrogativa das atribuições do enfermeiro. A inserção, o manuseio e a retirada do CV são atividades que compõem o processo de enfermagem, com cuidados que exigem complexidade técnica, conhecimentos de base científica e capacidade de tomar decisões imediatas^(4-5)^.

Aproximadamente 15% a 25% dos pacientes hospitalizados são expostos ao processo de CVD, e embora o procedimento de inserção do cateter exija técnica estéril, a complicação mais frequente é a ITU associada ao cateter (ITU-AC)^(3)^.

Evidências científicas apontam para um nível satisfatório de conhecimento quanto aos cuidados na inserção do CVD e à necessidade de revisão do uso diário^(6)^. Entretanto, fragilidades em relação ao conhecimento sobre as práticas que compõem o procedimento, especialmente nos quesitos indicações ao uso de CVD e cuidados na sua manutenção, ainda são destacadas.

A instituição participante do estudo possui protocolo de prevenção de ITU-AC e procedimento operacional padrão (POP) de passagem, manutenção e manuseio de CVD validados. No entanto, incidentes relacionados a esse processo assistencial continuam ocorrendo^(7)^. Nessa vertente, para qualificar o cuidado em relação ao uso do CVD, é necessário identificar as falhas e lacunas dessa prática assistencial desde a indicação até a retirada do dispositivo^(8)^. Ao avaliar um processo invasivo, que pode resultar em eventos adversos para os pacientes, por meio de uma ferramenta proativa, pretende-se identificar os pontos mais frágeis e propor melhorias para prevenir ou eliminar as falhas potenciais, aumentar a confiabilidade, a segurança e a qualidade do processo CVD.

O estabelecimento de uma cultura de comunicação aberta e de aprendizado a partir dos erros, com foco na não punição e na identificação de fatores de risco, pode possibilitar a redução desses incidentes relacionados à assistência à saúde^(9)^. A partir disso, torna-se possível implementar intervenções pontuais que promovam a mudança de hábitos dos profissionais e de suas expectativas sobre a necessidade, o uso seguro do CVD e o manejo dos eventos adversos, como a ITU-AC e o trauma uretral^(8)^.

Nessa perspectiva, objetivou-se analisar os riscos potenciais no processo de CVD em adultos.

## Método

### Delineamento do estudo

Trata-se de uma pesquisa exploratória e descritiva, que avaliou a melhoria da qualidade do processo assistencial de CVD, seguindo as recomendações do SQUIRE 2.0 para clareza e transparência na escrita^(10)^.

### Campo do estudo, local e período

A pesquisa foi desenvolvida em um hospital de ensino da região central do Rio Grande do Sul, Brasil. As unidades de pronto-socorro, internação de clínica médica e cirúrgica e unidade de tratamento intensivo foram os principais cenários assistenciais de cuidado ao paciente adulto elencados para desenvolver o estudo. A coleta de dados foi realizada entre agosto e dezembro de 2022.

### População, critérios de seleção e definição dos participantes

Foram convidados a participar da pesquisa os profissionais de saúde que realizavam assistência direta ao paciente adulto e eram membros do Núcleo de Segurança do Paciente e da Comissão de Controle de Infecções Relacionadas à Assistência à Saúde, totalizando 40 profissionais. Foram aplicados critérios de inclusão como pertencer à área de cuidado ao adulto, estar envolvido no processo assistencial elencado e trabalhar no mínimo a seis meses ou mais na instituição. Os critérios de exclusão foram estar afastado das atividades profissionais no momento da coleta de dados, licença saúde, especial ou férias.

A amostra foi não probabilística e por conveniência. Após o convite e aplicação dos critérios de seleção, o grupo de trabalho (GT) multidisciplinar foi formado por dez profissionais de saúde, os quais confirmaram a disponibilidade para desenvolvimento do método^(1)^. Destes, quatro enfermeiros assistenciais da área de cuidado ao adulto, um médico infectologista e intensivista, um técnico de enfermagem da unidade de tratamento intensivo adulto, dois enfermeiros do serviço de controle de infecção, um enfermeiro do setor de qualidade e o gestor de riscos assistenciais do hospital.

### Coleta e obtenção dos dados

Foram aplicadas as sete etapas preconizadas pelo método FMEA^(1)^. A *análise do sistema* ocorreu em três etapas (planejamento e preparação; análise da estrutura e de função). No planejamento e preparação (etapa 1), foi realizado, pela pesquisadora, um cronograma de prazos e tarefas com o GT, elencando ferramentas (*brainstorming*, fluxograma e diagrama de Ishikawa), formulários e tabelas de apoio preconizados pela análise proativa de riscos^(1)^. Já a análise da estrutura e de função (etapas 2 e 3) foi realizada pelo GT, por meio da elaboração de um fluxograma do processo com a descrição das falhas em potencial (Figura 1).

A *análise de falha e mitigação do risco* ocorreu em outras três etapas (análise das falhas, análise do risco e otimização). No estabelecimento da cadeia de falhas (etapa 4), o GT utilizou questionamentos para identificar o MPF, o EPF e a CPF. Para a análise de risco (etapa 5), obteve-se os índices de Severidade do EPF e de Ocorrência da CPF. Para a otimização (etapa 6) foi realizada pelo GT a atribuição de responsabilidades e prazos para implementação das ações (Figura 1).

Por fim, na *comunicação do risco* (etapa 7), foi realizada a definição de conteúdo da documentação dos resultados, conclusões e análise pelo GT. Neste momento final, o Número de Prioridade de Risco (NPR) é utilizado pelo GT para priorizar as falhas com alto risco e identificar as ações necessárias para minimizar seus impactos (Figura 1).

**Figura 1-:** Etapas percorridas pelo Método FMEA*. Santa Maria, RS, Brasil, 2023

Método FMEA*						
a. Análise do sistema			**b. Análise de falha e mitigação do risco**			**c. Comunicação do risco**
*Etapa 1. Planejamento e preparaçã* o	***Etapa 2. Análise da estrutura***	***Etapa 3. Análise de função***	***Etapa 4. Análise das Falhas***	***Etapa 5. Análise de Risco***	***Etapa 6. Otimização***	***Etapa 7. Documentação dos resultados e conclusões e análise***
*Plano do Projeto* (5T): *InTent* (Intenção), *Timing* (Prazo), *Team* (Equipe), *Tasks* (tarefas), *Tools* (Ferramentas)	Árvore da Estrutura e Função ou equivalente: *fluxograma de processo*	*Estabelecimento da cadeia de falhas*	*Atribuição dos Controles de Prevenção das Causas da Falha e dos Modos Potenciais de Falha*	*Atribuição de responsabilidades* e *prazos* para implementação das ações	*Definição* do conteúdo da *documentação*
*Passo 1:* *InTent* (Intenção): objeto desta pesquisa *Timing* (Prazo): agosto a dezembro de 2022 *Team* (Equipe): grupo de trabalho multidisciplinar *Tasks* (tarefas): encontros *Tools* (Ferramentas): fluxograma, *brainstormin* g e diagrama de Ishikawa	*Passo 2:* Construção, e avaliação, do fluxograma e descrição das falhas em potencial	*Passo 3:* Identificar o Modo Potencial de Falha (O que pode dar errado?) *Passo 4:* Identificar o Efeito Potencial da Falha (O que acontece? Quais seriam as consequências da falha?) *Passo 5:* Identificar a Causa Potencial de Falha (Por que a falha acontece?)	*Passo 4.1:* Obter índices de Severidade (Severidade do Efeito Potencial de Falha) *Passo 5.1:* Obter índices de Ocorrência (Probabilidade de Ocorrência da Causa Potencial de Falha) *Passo 6:* Determinar as medidas atuais de controles existentes e/ou planejados e pontuação das falhas *Passo 6.1:* Obter índices de Detecção (Probabilidade de Detecção da Falha) *Passo 7:* Calcular o NPR ^†^ (Severidade do efeito x Ocorrência da Causa Potencial de falha x Detecção da falha)	*Passo 8:* utilizar o NPR ^†^ para priorizar as falhas com alto risco *Passo 9:* Identificar as ações necessárias para reduzir riscos

Fonte: Adaptado^(2)^; *FMEA = Análise de Modo e Efeitos de Falha; ^†^NPR = Número de Prioridade de Risco

### Organização, análise e apresentação dos dados

A modelagem do processo foi realizada pelo *software* livre *Bizagi Modeler* ^®^ (versão 3.4.0.062/2019), que permitiu desenhar, diagramar, documentar e publicar os processos, utilizando a notação *Business Process Modeling Notation* (BPMN).

Todos os dados provenientes das reuniões foram transcritos para um banco eletrônico do programa *Microsoft Excel* ^®^. Após a validação da digitação, mediante dupla conferência do banco de dados pelo GT, foram geradas as análises, conforme descritas na Figura 1, e os relatórios.

### Aspectos éticos

A pesquisa foi registrada e aprovada pelo Comitê de Ética em Pesquisa da Universidade Federal de Santa Maria sob número 58032622.6.0000.5346.

## Resultados

O GT foi composto por dez profissionais de saúde, sendo: oito enfermeiros, um médico e um técnico de enfermagem. Houve predominância de profissionais de saúde do sexo feminino (n=9; 90%), com vínculo trabalhista conforme a Consolidação das Leis do Trabalho (CLT) (n=7; 70%), que exercem suas atividades laborais no turno da tarde (n=4; 40%) e com mestrado em ciências da saúde (n=6; 90%).

Para a aplicação do método FMEA, o GT desenvolveu 20 encontros, com duração de duas horas cada, totalizando 40 horas. Para realizar a discussão do subprocesso 1, foram necessários três encontros; para os subprocessos 2 e 4, foram utilizados quatro encontros; e para o subprocesso 3, foram necessários nove encontros.

Por meio das informações reunidas nos *brainstormings* realizados, o GT realizou o mapeamento do processo de CVD. Após, foram descritas quatro etapas-chave, sendo denominadas como subprocessos 1) Indicações para CVD, 2) Intervenção – passagem de CVD, 3) Manutenção e/ou troca do CVD e 4) Retirada do CVD (Figura 2). Os desdobramentos desses subprocessos foram descritos como atividades e tarefas.

**Figura 2-:** Processo de cateterismo vesical de demora. Santa Maria, RS, Brasil, 2023

Foram mapeados 4 subprocessos, 12 atividades e 70 tarefas. Emergiram três categorias profissionais com atribuições, competências e responsabilidades. O médico começa os subprocessos 1) Indicações para CVD e 4) Retirada de CVD, e o enfermeiro permeia os subprocessos 2) Intervenção – passagem de CVD e 3) Manutenção e/ou troca de CVD com auxílio do técnico de enfermagem.

Quanto aos modos, as causas e os efeitos potenciais de falha no processo de CVD, foram identificados 55 modos potenciais de falha, 92 causas potenciais de falhas e 40 efeitos potenciais de falha.

No subprocesso 1) Indicações para CVD, foram contabilizados 6 (10%) modos potenciais de falhas, 14 (15%) causas potenciais de falhas e 4 (10%) efeitos potenciais de falhas. Destacou-se o MPF *desconhecer as indicações para CVD* com alta Prioridade de Ação (PA). Apresentando a CPF *ausência da indicação de inserção no protocolo de prevenção de infecção do trato urinário (ITU-AC)* tendo como EPF a *cateterização desnecessária*.

O subprocesso 2) Intervenção – passagem de CVD evidenciou o maior número de atividades e tarefas, consequentemente apresentou o maior número de modos, causas e efeitos contabilizados. Sendo 43 (78%) modos potenciais de falha, 71 (78%) causas potenciais de falhas e 26 (66%) efeitos potenciais de falhas.

Neste subprocesso, o GT abordou as tarefas necessárias para que o procedimento de enfermagem consiga alcançar a indicação a qual se propõe. O MPF *não realizar a higiene do meato urinário* apresentou alta PA, apontando como causas potenciais de falhas *não conhecer o POP de inserção de CVD* e *não se importar com a higiene do meato urinário (saber a técnica e não realizar)* como principais contribuintes para o EPF *risco de ITU-AC; quebra de técnica asséptica*.

A atividade relacionada à Higienização das Mãos (HM) permeou o subprocesso 2, obtendo alta PA em dois modos potenciais de falhas relacionados à *HM inadequada - seja pela técnica e/ou tempo inadequado(s); pela perda de oportunidades para HM* e *não higienizar as mãos,* tendo como causas potenciais de falhas a *falta de acesso à infraestrutura (pias longe dos leitos), sobrecarga de trabalho; múltiplas tarefas*, *desinteresse em conhecer o protocolo de higienização das mãos disponível na intranet do hospital – não se importar com a HM*, *falta de acesso à infraestrutura (falta de dispensadores de solução alcoólica à beira dos leitos)* e *desinteresse em higienizar as mãos* sendo contribuintes para os efeitos potenciais de falhas relacionados ao ato de *não higienizar as mãos - aumentar o risco de Infecções Relacionadas à Assistência à Saúde (IRAS)* e *baixa adesão à higiene das mãos - aumentar o risco de IRAS.*

Outro MPF reportado com alta PA foi *não ter mesa de apoio auxiliar para o procedimento de inserção do CVD*, tendo como CPF a *falta de mesa ou carrinho auxiliar* e contribuindo para o EPF *risco de contaminação dos materiais, por estarem em local inadequado (por exemplo, cama do paciente)*.

No subprocesso 3) Manutenção e/ou troca de CVD, foram contabilizados três (5%) modos potenciais de falhas, três (3%) causas potenciais de falhas e três (7%) efeitos potenciais de falhas. Destacaram-se os modos potenciais de falhas *fixar a sonda foley com produto inadequado (por exemplo: micropore ou filme não estéril)* e *não fixar a sonda foley* com alta PA, respectivamente. Tendo como principais causas potenciais de falhas a *falta de fixador – produto adequado para a rotina* e *não fixar a sonda foley por esquecimento* ocasionando os efeitos potenciais de falhas *lesão de pele por dispositivo* ou *sangramento ou escarificação da uretra – risco de tração da sonda foley – risco de ITU-AC pela tração do sistema coletor.*

Corroborando, foi evidenciado que o MPF *não ter auxílio durante o procedimento de CVD* permeou os subprocessos 2 e 3, apresentando como principais causas potenciais de falhas a *falta de profissionais para compor a equipe de enfermagem,* s*obrecarga de trabalho; múltiplas tarefas*, *técnico de enfermagem não auxiliar o enfermeiro responsável pela passagem do CVD*, sendo os principais contribuintes para o EPF *risco de ITU-AC; quebra de técnica asséptica*.

No subprocesso 4) Retirada de CVD, foram contabilizados três (5%) modos potenciais de falhas, quatro (4%) causas potenciais de falhas e sete (17%) efeitos potenciais de falhas. Nenhum MPF apresentou alta PA, pois o subprocesso apresentou atividades discutidas pelo GT no subprocesso 2.

Porém, embora os modos potenciais de falhas relacionados ao subprocesso 4 tenham apresentado baixa PA, reportamos o MPF *não desinsuflar adequadamente o balonete da sonda foley* tendo como CPF *material sem qualidade* contribuindo para os efeitos potenciais de falhas relacionados ao *trauma uretral*, *não conseguir remover a sonda foley do meato urinário* ou *balonete estourar e pedaços de material (látex) permaneceram na bexiga do paciente.*

As causas potenciais das falhas (n=92; 100%) foram inseridas no diagrama de Ishikawa, sendo reclassificadas e categorizadas em causas relacionadas ao uso de equipamento (n=21; 23%), comportamento/habilidade/atitude (n=18; 19%), educação e treinamento (n=17; 18%), sociais ou de trabalho em time (n=12; 13%), atividade/tarefa (n=11; 12%), paciente (n=5; 6%), ambiente de trabalho (n=5; 6%) e comunicação (n=3; 3%).

## Discussão

A classificação dos pacientes em termos de presença (ou não) e uso apropriado do CVD pode ser baseada em guias^(3,11)^. Neste ínterim, verificou-se que o protocolo de prevenção de ITU-AC da instituição pesquisada não contemplava as principais indicações de inserção do cateter vesical. Apesar de a avaliação clínica para CVD ser soberana, as indicações^(3)^ podem nortear boas práticas, destacando os riscos e benefícios para auxiliar os médicos na tomada de decisão.

Pesquisadores^(12-13)^ destacam que enfermeiros e médicos não estão familiarizados com as indicações para CVD. Estudo de coorte com 388 pacientes evidenciou que a cada quatro pacientes, um foi submetido à colocação de uma sonda vesical [24,7% (n=96); Intervalo de Confiança 95% (IC95%): 20% a 29%], sendo que em 36,5% (IC95%: 33% a 48%) dos casos não apresentavam critérios clínicos para tal procedimento^(12)^. O uso inapropriado de CVD é mais comum em enfermarias clínicas do que em enfermarias cirúrgicas, sendo que o uso inapropriado do dispositivo ocorreu em 14 (10,4%) dos 134 pacientes cirúrgicos, em comparação com 105 (32,4%) de 324 pacientes clínicos, uma diferença de 22% (IC95%: 14,7-29,2; p<0,001)^(13)^.

Quanto à cateterização desnecessária, estudos^(14-15)^ com profissionais de saúde objetivaram realizar intervenções para diminuir a inserção de CV e, consequentemente, complicações infecciosas e não infecciosas. Estes aplicaram pacotes com medidas para alinhar os conhecimentos de médicos e enfermeiros sobre indicações de uso de cateteres. Foram incluídas situações específicas em que o cateterismo urinário é desnecessário e ações educativas que variaram com o tempo e metodologia. Um conjunto simples de medidas aumentou a utilização de CV com indicações baseadas em evidências e resultou em mudança na prática relacionada ao uso contínuo do dispositivo. As taxas de ITU-AC permaneceram em níveis baixos. No entanto, não teve impacto nas complicações não infecciosas^(14-15)^.

Ao abordar o preparo do ambiente para o procedimento de inserção do CVD, uma avaliação do leito do paciente que será submetido ao procedimento deverá ser realizada. Recomenda-se que a avaliação do leito inclua a cama do paciente (travesseiro, coxins, lençóis), materiais médicos hospitalares disponíveis, como mesa de apoio, foco de luz e biombos para garantir a privacidade do paciente.

Quanto ao preparo do paciente para o procedimento, no que tange à higiene do meato urinário, não há consenso na literatura sobre a escolha de produtos para higiene, e as diretrizes diferem em suas recomendações. A diretriz americana^(3)^ não fornece recomendação, afirmando que o problema permanece sem solução e apresenta lacuna de pesquisas sobre o uso de soluções antissépticas. Já a diretriz inglesa^(11)^ recomenda o uso de solução salina para higiene do meato uretral antes da inserção do cateter.

A HM permeou o subprocesso 2. No ambiente hospitalar, a HM é imprescindível para evitar danos relacionados à assistência à saúde. Necessita de maior comprometimento por parte de todos os envolvidos. Nesse quesito, intervenções realizadas por gestores para conformidade da infraestrutura e insumos (pias, torneiras, dispensadores, papel toalha) são importantes para garantir condições necessárias aos profissionais de saúde que prestam assistência direta ao paciente^(16)^.

A segurança e a qualidade do cuidado no processo de enfermagem relacionado a CVD não estão relacionadas somente com a qualificação profissional e com as medidas de prevenção. Existem outros fatores que podem afetar este processo assistencial, como recursos, materiais suficientes e de qualidade, bem como uma estrutura adequada para a execução do procedimento.

Ao abordar o procedimento de inserção, o GT destacou as competências do enfermeiro e as atribuições do técnico de enfermagem em situações de necessidade de suporte para realização do procedimento de inserção do CVD. Nesse sentido, é necessário que o profissional enfermeiro tenha conhecimento científico e habilidade técnica, buscando um equilíbrio entre a segurança do paciente e custo-benefício^(17-18)^.

No que tange à prática clínica, o *guideline* da *European Association of Urology Nurses* (EAUN) apresenta diretrizes, baseada em evidências, para apoiar uma prática de inserção do CVD uniforme em adultos^(19-20)^. Um estudo sueco avaliou o impacto das diretrizes da EAUN sobre técnica asséptica durante a cateterização em dois hospitais. A conformidade do procedimento com técnica asséptica foi associada às equipes de cirurgia e cardiologia (*Odds ratio* (OR): 2,35; IC95%: 1,69 - 3,27), ao uso de “kit” estéril para cateterismo (OR: 2,06; IC95%: 1,42 - 2,97), ao uso de campos estéreis para a área de inserção (OR: 1,91; IC95%: 1,24 - 2,96) e ao uso do termo “técnica estéril” para a inserção do CV (OR: 1,64; IC95%: 1,11 - 2,43). O resultado mostrou que 55 a 74% dos enfermeiros praticaram uma ou mais precauções que garantiram a técnica asséptica da inserção de CV, demonstrando uma lacuna entre as diretrizes da EAUN e o desempenho real. Foram mencionadas estratégias de melhorias relacionadas ao monitoramento da técnica estéril durante a inserção do cateter, para possibilitar a redução do risco de contaminação e implementação de um protocolo de inserção de CV contínuo com a presença de duas pessoas com vistas à redução de ITU-AC^(21)^.

Neste ínterim, não ter auxílio do técnico de enfermagem durante o procedimento de inserção do CVD destaca a necessidade de cooperação durante a execução do procedimento de inserção. Corroborando este achado, a implementação do protocolo de inserção com dois profissionais indicou que isso, somado a outras estratégias, como a aplicação de lista de verificação de tarefas, diminuiu o risco de ITU-AC dos pacientes estudados^(22)^.

No Brasil, o parecer normativo para atuação da equipe de enfermagem em sondagem vesical estabelece diretrizes visando à efetiva segurança do paciente submetido ao procedimento^(4)^. Descreve que a passagem do CV é privativa do enfermeiro e apresenta ações que devem ser desenvolvidas pela equipe de enfermagem durante a execução do procedimento.

Porém, destaca-se a necessidade de legislações que reportem a presença obrigatória do técnico de enfermagem, no que tange a responsabilidades relacionadas à preparação do material e do ambiente, ao posicionamento do paciente, à abertura do material estéril, ao destino dos materiais utilizados e aos resíduos gerados e, quando necessário, ao encaminhamento para laboratório do material coletado para exames. Essas responsabilidades poderão contribuir para reduzir a ocorrência de incidentes, fortalecendo a segurança do paciente, com rigor técnico-científico e trabalho em equipe.

Quanto à manutenção do CVD, a fixação do cateter é uma prática reconhecida como um aspecto importante e essencial para a manutenção do dispositivo. Consiste em utilizar um dispositivo para fixar um cateter *foley* urinário na região interna da coxa (mulher) ou na parte inferior do abdome (homem) do paciente. A forma inadequada de fixar pode levar a complicações como remoção inadvertida, lesões na pele, trauma ou erosão uretral^(23)^.

Um estudo canadense verificou uma prevalência geral de 18% (8/44) de fixação do cateter. Destes, sete foram fixados corretamente, e o principal método de fixação foi um adesivo comercial (6/8; 75%)^(24)^. A lesão de pele por dispositivo está relacionada à presença prolongada do cateter e/ou à falta de fixação do cateter; ela pode causar desconforto, irritação da pele e do epitélio uretral, eritema na borda do meato, passando pela perda total da espessura do tecido, até a clivagem peniana completa, desfiguração peniana e disfunção sexual e urinária^(25)^.

As diretrizes^(3,19,25)^ recomendam que, se um cateter permanente não puder ser evitado, ele deve ser fixado ao corpo do paciente para prevenir a tração do cateter e pressão sobre o meato uretral. A recomendação está baseada em um nível baixo de evidência. Portanto, são necessários estudos prospectivos para estabelecer diretrizes baseadas em evidências. Os cuidados relacionados à fixação da sonda vesical são de responsabilidade da equipe de enfermagem e devem incluir ações para a prevenção de lesões. Nesse contexto, conscientizar a equipe quanto à importância de ações para prevenção de lesões por pressão associadas ao dispositivo invasivo é o primeiro passo.

Depois de alcançar o seu propósito, o CV deve ser removido. O aprisionamento do CV pode ocorrer devido ao canal de insuflação defeituoso, ao mau funcionamento ou à cristalização do fluido dentro do balonete. As causas de cateteres *foley* retidos e o método para lidar com cada incidente podem variar^(26)^.

Ao percorrer o processo de CV contínuo, é possível perceber dicotomia entre a prática, o ensino e as evidências científicas. Nesse sentido, verifica-se que a abordagem de aspectos relacionados à inovação e tecnologia nas publicações associadas ao gerenciamento de riscos do processo CV é escassa, assim como em outros procedimentos de enfermagem realizados rotineiramente^(27)^.

Em contraponto, ações para melhorar a prática clínica e reduzir incidentes com eventos adversos têm sido investigadas. Estas ações contemplam principalmente a prevenção de fatores de riscos modificáveis para ITU-AC e trauma uretral^(7,28-30)^. Programas de melhoria de qualidade para reduzir o uso de CVD utilizando pacotes de intervenções baseadas em evidências, com foco em evitar o uso desnecessário, bem como promover inserção, técnica e manutenção do cateter adequadas, têm sido descritos na literatura^(31-32)^.

As situações e razões para omissão do cuidado, que é quando “o profissional não consegue executar a ação certa quer seja na fase de planejamento ou na fase de execução”^(33)^, permearam a análise de risco do processo de CVD. Esses achados estão em concordância com a literatura que destaca as razões mais frequentes para a omissão dos cuidados de enfermagem relacionadas a recursos humanos (escassez da força de trabalho), recursos materiais e aumento inesperado de pacientes^(34-35)^.

Nesse quesito, o GT reportou a normalização do desvio, termo utilizado para um fenômeno que acontece quando as pessoas de uma organização se tornam tão insensíveis que a prática desviante não parece mais errada. A insensibilidade ocorre diariamente e, muitas vezes, se perpetuam nas equipes ao longo dos anos, sem ter a devida importância até que fatores críticos se alinhem em direção ao incidente/dano^(36)^. Diante do exposto, não se importar com a higiene do meato urinário (saber a técnica e não realizar) é um exemplo de desvio da prática baseada em evidência e de violação de controle de infecção.

Cabe destacar que os profissionais de saúde não tomam decisões com a intenção de aumentar os riscos assistenciais e causar danos aos pacientes. Porém, o desvio ocorre devido à quebra de barreiras do processo ou direcionadores como tempo, custo, sobrecarga de trabalho e pressão dos colegas^(36)^. Nessa conjuntura, avaliar os achados desta pesquisa sob a ótica desses conceitos pode auxiliar a minimizar ou evitar a normalização de desvio e omissão do cuidado. Isso pode acontecer por meio de ações instituídas com transparência e ferramentas de melhoria de desempenho, para que a mudança e a prevenção dos desvios iniciem na linha de frente, mas sejam respaldadas pela gestão.

Quanto à falta de adesão às boas práticas, é importante observar e estudar os cenários de assistência, os processos, as relações interpessoais, para que se capte os motivos, ao considerar a complexidade do processo decisório e as diferentes percepções entre os profissionais de saúde.

O CVD é um tema multiprofissional, com responsabilidades compartilhadas que oscilam entre categorias profissionais. Quando necessário intervir para gerar melhorias, é importante que as medidas de sensibilização e comunicação de novas rotinas e padrões sejam direcionadas a toda a equipe de saúde, principalmente médicos e enfermeiros.

Espera-se que este estudo forneça subsídios para repensar os problemas e falhas relacionados aos processos assistenciais para além da enfermagem, com vistas a minimizar os riscos no cuidado e prevenir incidentes de segurança aos pacientes. Desse modo, o compromisso da liderança com a utilização do CVD, de forma restrita e racional, precisa ser visível além do grupo multiprofissional, abordando o paciente e acompanhante para pavimentar o caminho para a melhoria.

Quanto às limitações deste estudo, o escopo do FMEA realizado pelo GT limitou-se ao processo de CVD em adultos, contemplando os quatro subprocessos apresentados. Não foram incluídos no escopo subprocessos relacionados à transição do cuidado, à transferência e ao transporte de pacientes com CVD, com irrigação contínua ou lavagem vesical. Não foram abordados grupos de pacientes com faixa etárias específicas (pessoas idosas, gestantes, recém-nascidos, bebês e crianças), além do engajamento do paciente e familiar.

Devido ao GT desconhecer a ferramenta, a dificuldade enfrentada foi a sua utilização. A aplicação do método demandou atenção devido à complexidade dos conceitos e das cadeias de falhas. Porém, após o período de incorporação e adaptação, com a retomada do método, sempre que necessário, o FMEA possibilitou uma discussão coletiva com amplitude reflexiva sobre o fazer diário assistencial. Uma vez que os resultados relacionados à cadeia de falhas são inerentes a um hospital universitário, sugere-se ampliar a aplicação desta ferramenta para outras instituições.

## Conclusão

A análise proativa de riscos assistenciais realizada no ambiente hospitalar fortaleceu a aplicabilidade da ferramenta em um processo de saúde. Temática que carece de maior visibilidade para contribuir com a cultura de segurança em âmbito nacional.

Proporcionou ao GT a revisão do processo de CVD, aumentando a percepção dos riscos relacionados à segurança do paciente e discutindo ações para torná-lo mais seguro e qualificado. Foram mapeados 4 subprocessos, 12 atividades e 70 tarefas que evidenciaram as correlações entre os subprocessos/atividades, profissionais de saúde e informações. A análise proativa de riscos assistenciais do processo de CVD no ambiente hospitalar evidenciou 55 MPFs, 92 CPFs e 40 EPFs.

O subprocesso 2 apresentou em suas cadeias de falhas os maiores riscos que necessitam ser gerenciados proativamente. Destacou a posição estratégica dos enfermeiros na tomada de decisão para gestão de riscos relacionados à prática assistencial do processo de CV, desde a inserção e o uso contínuo até a remoção do dispositivo.

As principais recomendações sugeridas pelo GT contemplaram os modos potenciais de falha com alta PA e formaram uma lista para proporcionar o aprimoramento de resultados relacionados ao objeto do estudo. Contemplou ações relacionadas à implementação de discussões multidisciplinares para evitar o uso desnecessário de CVD; revisão do protocolo de prevenção de ITU-AC e do POP de inserção de CVD; institucionalização de lista de verificação de inserção e aplicação de pacote para manutenção e manuseio de CVD, acompanhado de um programa de capacitação com o envolvimento da academia. Por fim, recomendou um levantamento das necessidades de melhorias relacionadas à infraestrutura hospitalar, à qualificação do processo de aquisição de artigos médicos hospitalares e ao dimensionamento das equipes de enfermagem.

# Translation: Análisis del modo y efecto de la falla en el proceso de sondaje vesical de demora*

**DOI:** 10.1590/1518-8345.7589.4794

**Authors:** Caroline Zottele Piasentin Giacomini, Ana Elisa Bauer de Camargo Silva, Janete de Souza Urbanetto, Paulo Sousa, Tania Solange Bosi de Souza Magnago

**Affiliations:** 1 Universidade Federal de Santa Maria, Santa Maria, RS, Brasil; 2 Universidade Federal de Goiás, Faculdade de Enfermagem, Goiânia, GO, Brasil; 3 Pontifícia Universidade Católica do Rio Grande do Sul, Escola de Ciências da Saúde e da Vida, Porto Alegre, RS, Brasil; 4 Universidade NOVA de Lisboa, Escola Nacional de Saúde Pública, Lisboa, LX, Portugal

**Keywords:** Seguridad del Paciente, Gestión de Riesgos, Calidad de la Atención de Salud, Análisis de Modo y Efecto de Fallas en la Atención de la Salud, Atención de Enfermería, Catéteres Urinários

## Abstract

analizar los riesgos potenciales en el proceso de sondaje vesical de demora en adultos.

investigación exploratoria, descriptiva y evaluativa realizada en un hospital universitario. El grupo de trabajo estuvo compuesto por diez profesionales de la salud, ocho enfermeros, un médico y un técnico de enfermería. La construcción del diagrama de flujo y descripción de actividades se realizó mediante modelado del proceso. El análisis de los riesgos potenciales con base en el método Failure Mode and Effect Analysis.

se identificaron cuatro subprocesos para el proceso de sondaje vesical de demora. El análisis proactivo de los riesgos asistenciales relacionados con la revisión del proceso destacó 55 modos de falla potenciales, 92 causas de falla potenciales y 40 efectos de falla potenciales.

la aplicabilidad del método utilizado para revisar el proceso de sondaje vesical de demora en el ámbito hospitalario fortaleció el análisis proactivo de los riesgos relacionados con la salud, tema que requiere mayor visibilidad para contribuir a una cultura de seguridad en el ámbito nacional. Se destacó la posición estratégica de los enfermeros en la toma de decisiones para gestionar los riesgos relacionados con la práctica asistencial del proceso destacado, desde la inserción y el uso continuo hasta la retirada del dispositivo.

## Destacados

**(1)** Análisis proactivo de riesgos realizado en un proceso asistencial. **(1)** Modelado del proceso de sondaje vesical de demora. **(1)** Los enfermeros son estratégicos para gestionar el proceso de sondaje vesical de demora.

## Introducción

La gestión de riesgos asistenciales de salud es un aspecto esencial de la seguridad del paciente en los servicios, ya que busca identificar y tratar los riesgos a los que están expuestos los pacientes, los profesionales y las organizaciones de salud^(1)^. Desde esta perspectiva, el Análisis Modal de Fallas y Efectos (FMEA, por sus siglas en inglés, *Failure Mode and Effect Analysis*) es una herramienta que puede utilizarse de forma proactiva para prevenir incidentes de seguridad. Es adecuada y eficaz para gestionar riesgos de cualquier naturaleza^(1)^.

La aplicabilidad del FMEA se da de manera transversal, considerando los conceptos relacionados con la cadena de fallas, como el modo de falla potencial (MFP), la causa de falla potencial (CFP) y el efecto de falla potencial (EFP). Además, también se puede comprobar la Severidad del EFP, la probabilidad de Ocurrencia de la CFP y la probabilidad de Detección/Prevención del MFP que pueden afectar al paciente^(2)^.

Entre los procesos asistenciales que requieren gestión de riesgos, destaca el proceso de Sondaje Vesical de Demora (SVD). Una intervención invasiva de rutina, capaz de causar complicaciones relacionadas con la Infección del Tracto Urinario (ITU) y otras con daño al tracto urinario inferior, como dolor, trauma uretral, falso camino, fístula uretral, prostatitis, epididimitis^(3)^. El procedimiento consiste en insertar una sonda estéril hasta la vejiga, a través de la uretra, con el objetivo principal de facilitar el drenaje de la orina, manteniendo la vejiga sondada con una sonda conectada a un sistema estéril cerrado durante horas o días.

La sonda vesical (SV) es insertada, mantenida y retirada por enfermeros, mientras que los médicos son responsables de ordenar la inserción y la extracción. Cabe resaltar el proceso de SVD es prerrogativa de las funciones del enfermero. La inserción, la manipulación y la retirada de la SV son actividades que forman parte del proceso de enfermería, con cuidados que requieren complejidad técnica, conocimientos de base científica y capacidad de tomar decisiones inmediatas^(4-5)^.

Aproximadamente entre el 15% y el 25% de los pacientes hospitalizados están expuestos al proceso de SVD, y, aunque el procedimiento de inserción de la sonda requiere una técnica estéril, la complicación más frecuente es la ITU asociada a catéter (ITU-CA)^(3)^.

Evidencias científicas apuntan a un nivel satisfactorio de conocimiento en cuanto a los cuidados en la inserción de SVD y la necesidad de revisar su uso diario^(6)^. Sin embargo, aún se destacan debilidades en relación con el conocimiento sobre las prácticas que componen el procedimiento, especialmente en cuanto a las indicaciones de uso de SVD y los cuidados en su mantenimiento.

La institución que participa en el estudio cuenta con un protocolo para la prevención de ITU-AC y un procedimiento operativo estándar (POE) validado para el paso, mantenimiento y manipulación de SVD. Sin embargo, siguen ocurriendo incidentes relacionados con este proceso asistencial^(7)^. En ese sentido, para calificar el cuidado en relación con el uso de SVD, es necesario identificar las fallas y brechas en esta práctica asistencial desde la indicación hasta la retirada del dispositivo^(8)^. Al evaluar un proceso invasivo, que puede resultar en eventos adversos para los pacientes, mediante una herramienta proactiva, se busca identificar los puntos más débiles y proponer mejoras para prevenir o eliminar potenciales fallas, aumentar la confiabilidad, la seguridad y la calidad del proceso de SVD.

Establecer una cultura de comunicación abierta y de aprendizaje de los errores, con foco en la no sanción y en la identificación de factores de riesgo, puede permitir la reducción de estos incidentes relacionados con la asistencia a la salud^(9)^. A partir de esto, se hace posible implementar intervenciones específicas que promuevan el cambio de hábitos de los profesionales y de sus expectativas en relación con la necesidad, el uso seguro de SVD y el manejo de los eventos adversos, como la ITU-AC y el trauma uretral^(8)^.

Desde esta perspectiva, el objetivo fue analizar los riesgos potenciales en el proceso de SVD en adultos.

## Método

### Diseño del estudio

Se trata de una investigación exploratoria y descriptiva, que evaluó la mejora en la calidad del proceso asistencial de SVD, siguiendo las recomendaciones del SQUIRE 2.0 de claridad y transparencia en la redacción^(10)^.

### Campo de estudio, ubicación y período

La investigación se desarrolló en un hospital docente de la región central de Rio Grande do Sul, Brasil. Las unidades de urgencias, hospitalización de clínica médica y quirúrgica y la unidad de cuidados intensivos fueron los principales escenarios asistenciales de cuidado al paciente adulto seleccionados para desarrollar el estudio. La recopilación de datos se realizó entre agosto y diciembre del 2022.

### Población, criterios de selección y definición de participantes

Se invitó a participar en la investigación a profesionales de la salud que brindaban atención directa a pacientes adultos y eran miembros del Núcleo de Seguridad del Paciente y de la Comisión de Control de Infecciones Relacionadas con la Asistencia a la Salud, totalizando 40 profesionales. Se aplicaron criterios de inclusión como pertenecer al área de cuidado al adulto, participar en el proceso asistencial enumerado y trabajar al menos seis meses o más en la institución. Se excluyeron aquellos que no estaban ejerciendo las actividades profesionales en el momento de la recopilación de datos, ya sea por estar de baja por enfermedad, licencia especial o vacaciones.

La muestra fue no probabilística y por conveniencia. Tras la invitación y aplicación de los criterios de selección, el grupo de trabajo (GT) multidisciplinario quedó conformado por diez profesionales de la salud, quienes confirmaron su disponibilidad para el desarrollo del método^(1)^. De ellos, cuatro enfermeros asistenciales del ámbito de cuidado al adulto, un médico infectólogo e intensivista, un técnico de enfermería de la unidad de cuidados intensivos para adultos, dos enfermeros del servicio de control de infecciones, un enfermero del sector de calidad y el gestor de riesgos asistenciales del hospital.

### Recopilación y obtención de los datos

Se aplicaron los siete pasos recomendados por el método FMEA^(1)^. El análisis del sistema se produjo en tres etapas (planificación y preparación; análisis de la estructura y de función). En la planificación y preparación (paso 1), la investigadora elaboró un cronograma de plazos y tareas con el GT, enumerando herramientas (*brainstorming*, diagrama de flujo y diagrama de Ishikawa), formularios y tablas de apoyo recomendados por el análisis proactivo de riesgos^(1)^. A su vez, el análisis de la estructura y de función (pasos 2 y 3) fue realizado por el GT, mediante la elaboración de un diagrama de flujo del proceso con la descripción de fallas potenciales (Figura 1).

El análisis de falla y mitigación del riesgo se produjo en otros tres pasos (análisis de las fallas, análisis del riesgo y optimización). Al establecer la cadena de fallas (paso 4), el GT utilizó preguntas para identificar el MFP, EFP y CFP. Para el análisis de riesgo (paso 5), se obtuvieron los índices de Severidad del EFP y Ocurrencia de la CFP. Para la optimización (paso 6), el GT asignó responsabilidades y plazos para la implementación de las acciones (Figura 1).

Por último, en la comunicación de riesgo (paso 7), se llevó a cabo la definición del contenido de la documentación de los resultados, conclusiones y análisis por el GT. En esta etapa final, el GT utilizó el Número de Prioridad de Riesgo (NPR) para priorizar las fallas con alto riesgo e identificar las acciones necesarias para minimizar sus impactos (Figura 1).

**Figura 1–:** Pasos cubiertos por el Método FMEA*. Santa Maria, RS, Brasil, 2023

Método FMEA*						
a. Análisis del sistema			**b. Análisis de falla y mitigación del riesgo**			**c. Comunicación del riesgo**
*Paso 1. Planificación y preparación*	*Paso 2. Análisis de la estructura*	*Paso 3. Análisis de función*	*Paso 4. Análisis de fallas*	*Paso 5. Análisis de riesgos*	*Paso 6. Optimización*	*Paso 7. Documentación de los resultados y conclusiones y análisis*
*Plan del proyecto* (5T): *InTent* (intención), *Timing* (plazo), *Team* (equipo), *Tasks* (tareas), *Tools* (herramientas)	Árbol de estructura y función o equivalente: *diagrama de flujo del proceso*	*Establecer la cadena de fallas*	*Asignación de Controles de Prevención de las Causas de Falla y de los Modos Potenciales de Falla*	*Asignación de responsabilidades y plazos* para implementación de las acciones	*Definición* del contenido de la *documentación*
*Paso 1* : *InTent* (intención): objeto de esta investigación *Timing* (plazo): agosto a diciembre del 2022 *Team* (equipo): grupo de trabajo multidisciplinario *Tasks* (tareas): reuniones *Tools* (herramientas): diagrama de flujo, *brainstorming* y diagrama de Ishikawa	*Paso 2:* Construcción y evaluación del diagrama de flujo y descripción de fallas potenciales	*Paso 3:* Identificar el Modo Potencial de Falla (¿Qué podría salir mal?) *Paso 4:* Identificar el Efecto Potencial de Falla (¿Qué sucede? ¿Cuáles serían las consecuencias de la falla? *Paso 5:* Identificar la Causa de Falla Potencial (¿Por qué ocurre la falla?)	*Paso 4.1:* Obtener índices de Severidad (Severidad del Efecto de Falla Potencial) *Paso 5.1:* Obtener índices de Ocurrencia (Probabilidad de Ocurrencia de la Causa de Falla Potencial) *Paso 6:* Determinar las medidas actuales de controles existentes o planificados y puntuación de las brechas *Paso 6.1:* Obtener índices de Detección (Probabilidad de Detección de la Falla) *Paso 7:* Calcular el NPR ^†^ (Severidad del efecto x Ocurrencia de la Causa Potencial de Falla x Detección de la falla)	*Paso 8:* utilizar el NPR ^†^ para priorizar las fallas con alto riesgo *Paso 9:* Identificar las acciones necesarias para reducir los riesgos

Fuente: Adaptado^(2)^; *FMEA = Análisis Modal de Fallas y Efectos; ^†^NPR = Número de Prioridad de Riesgo

### Organización, análisis y presentación de los datos

El modelado del proceso se realizó utilizando el *software* libre *Bizagi Modeler* ^®^ (versión 3.4.0.062/2019), que permitió diseñar, diagramar, documentar y publicar los procesos, utilizando la notación *Business Process Modeling Notation* (BPMN).

Todos los datos provenientes de las reuniones se transcribieron a una base electrónica en el programa Microsoft Excel^®^. Tras la validación de la introducción manual de datos, mediante doble verificación de la base de datos por parte del GT, se generaron los análisis, como se describen en la Figura 1, y los informes.

### Aspectos éticos

La investigación fue registrada y aprobada por el Comité de Ética en Investigación de la Universidad Federal de Santa Maria bajo el número 58032622.6.0000.5346.

## Resultados

El GT estuvo integrado por diez profesionales de la salud, entre ellos: ocho enfermeros, un médico y un técnico de enfermería. Se observó predominio de profesionales de la salud mujeres (n=9; 90%), con contrato de trabajo según la Consolidación de Leyes del Trabajo (CLT) (n=7; 70%), que desempeñan sus actividades laborales en el turno de la tarde (n=4; 40%) y con título de maestría en ciencias de la salud (n=6; 90%).

Para aplicar el método FMEA, el GT desarrolló 20 reuniones, de dos horas de duración cada una, totalizando 40 horas. Para discutir el subproceso 1, se realizaron tres encuentros; para los subprocesos 2 y 4 se realizaron cuatro encuentros; y para el subproceso 3, se realizaron nueve reuniones.

Por medio de la información reunida en los brainstormings realizados, el GT realizó el mapeo del proceso de SVD. Posteriormente, se describieron cuatro pasos claves, los cuales se denominan subprocesos 1) Indicaciones para SVD, 2) Intervención – paso de SVC, 3) Mantenimiento o reemplazo del SVD y 4) Retirada del SVD (Figura 2). El desarrollo de estos subprocesos se describió como actividades y tareas.

**Figura 2:** – Proceso de sondaje vesical de demora. Santa Maria, RS, Brasil, 2023

Se mapearon 4 subprocesos, 12 actividades y 70 tareas. Surgieron tres categorías profesionales con deberes, competencias y responsabilidades. El médico inicia los subprocesos 1) Indicaciones de SVD y 4) Retirada de SVD, y el enfermero permea los subprocesos 2) Intervención – paso de SVD y 3) Mantenimiento o reemplazo de SVD con ayuda del técnico de enfermería.

En cuanto a los modos, las causas y los efectos de falla potenciales en el proceso de SVD, se identificaron 55 modos de falla potenciales, 92 causas de falla potenciales y 40 efectos de falla potenciales.

En el subproceso 1) Indicaciones de SVD, se registraron 6 (10%) modos de fallas potenciales, 14 (15%) causas de fallas potenciales y 4 (10%) efectos de fallas potenciales. Destacó el MFP desconocer las indicaciones de SVD con alta Prioridad de Acción (PA). Que presenta la CFP ausencia de indicación para inserción en el protocolo de prevención de infección del tracto urinario (ITU-AC) y tiene como EFP el sondaje innecesario.

El subproceso 2) Intervención – paso de SVD presentó el mayor número de actividades y tareas, presentando consecuentemente el mayor número de modos, causas y efectos contabilizados. Hay 43 (78%) modos de fallas potenciales, 71 (78%) causas de fallas potenciales y 26 (66%) efectos de fallas potenciales.

En este subproceso, el GT abordó las tareas necesarias para que el procedimiento de enfermería logre alcanzar la indicación a la que se propone. El MFP no realiza la higiene del meato urinario presentó alta PA, señalando como causas de fallas potenciales no conocer el POE para la inserción de SVD y no preocuparse por la higiene del meato urinario (conocer la técnica y no realizarla) como contribuyentes principales al EFP riesgo de ITU-AC; incumpliendo la técnica aséptica.

La actividad relacionada con la Higienización de las manos (HM) permeó el subproceso 2 y obtuvo una PA alta en dos modos de fallas potenciales relacionados con la HM inadecuada - ya sea debido a la técnica o al tiempo inadecuado(s); debido a la pérdida de oportunidades de HM y no higienizarse las manos, tuvo como causas de fallas potenciales la falta de acceso a la infraestructura (lavabos lejos de las camas), sobrecarga de trabajo; múltiples tareas, desinterés por conocer el protocolo de higienización de las manos disponible en la intranet del hospital – no preocuparse por la HM, falta de acceso a la infraestructura (falta de dispensadores de solución alcohólica al lado de las camas) y desinterés por higienizarse las manos, lo que contribuye a los efectos de fallas potenciales relacionados con el acto de no higienizarse las manos, aumentar el riesgo de Infecciones Relacionadas con la Asistencia a la Salud (IRAS) y baja adhesión a la higiene de las manos, aumentar el risco de IRAS.

Otro MFP reportado con PA alta fue no tener una mesa de apoyo auxiliar para el procedimiento de inserción de la SVD, teniendo como CFP la falta de mesa o carrito auxiliar y contribuyendo al EFP riesgo de contaminación de los materiales, por estar en un lugar inadecuado (por ejemplo, la cama del paciente).

En el subproceso 3) Mantenimiento o reemplazo de SVD, se contabilizaron tres (5%) modos de falla potenciales, tres (3%) causas de falla potenciales y tres (7%) efectos de falla potenciales. Destacaron los modos de falla potenciales fijar la sonda foley con un producto inadecuado (por ejemplo, microporo o película no estéril) y no fijar la sonda foley con presión arterial alta, respectivamente. Tuvo como principales causas de fallas potenciales la falta de fijador —producto adecuado para la rutina y no fijar la sonda foley por olvido, lo que ocasiona los efectos potenciales de fallas del tipo lesión en la piel por dispositivo o sangrado o escarificación de la uretra— risco de tracción de la sonda foley —riesgo de ITU-AC debido a la tracción del sistema de recolección.

Lo que corroborando que el MFP no recibe ayuda durante el procedimiento de SVD permeó los subprocesos 2 y 3, y presentó como principales causas de fallas potenciales la falta de profesionales para componer el equipo de enfermería, sobrecarga de trabajo; múltiples tareas, el técnico de enfermería no ayuda al enfermero responsable del paso del SVD, y los principales contribuyentes para el EFP riesgo de ITU-AC; incumplimiento de la técnica aséptica.

En el subproceso 4) Retirada de SVD, Se contabilizaron tres (5%) modos de falla potenciales, cuatro (4%) causas de falla potenciales y siete (17%) efectos de falla potenciales. Ningún MFP presentó PA alta, ya que el subproceso presentó actividades discutidas por el GT en el subproceso 2.

Sin embargo, aunque los modos potenciales de fallas relacionados con el subproceso 4 presentaron una baja PA, reportamos el MFP no desinflar adecuadamente el balón de la sonda Foley, teniendo como CFP el material de baja calidad, lo que contribuye a los efectos potenciales de fallas relacionados con el trauma uretral, la imposibilidad de retirar la sonda Foley del meato urinario o que el balón estalle y fragmentos de material (látex) queden en la vejiga del paciente.

Las causas de fallas potenciales (n=92; 100%) se incluyeron en el diagrama de Ishikawa, se reclasificaron y se categorizaron en causas relacionadas con el uso del equipo (n=21; 23%), comportamiento/habilidad/actitud (n=18; 19%), educación y entrenamiento (n=17; 18%), trabajo social o en equipo (n=12; 13%), actividad/tarea (n=11; 12%), paciente (n=5; 6%), ambiente de trabajo (n=5; 6%) y comunicación (n=3; 3%).

## Discusión

La clasificación de los pacientes en términos de presencia (o ausencia) y uso apropiado de SVD puede basarse en guías^(3,11)^. Mientras tanto, se constató que el protocolo de prevención de ITU-AC de la institución analizada no cubría las principales indicaciones para la inserción de la sonda vesical. Aunque la evaluación clínica para SVD es primordial, las indicaciones^(3)^ pueden orientar buenas prácticas, resaltando los riesgos y beneficios para ayudar a los médicos en la toma de decisiones.

Algunos investigadores^(12-13)^ resaltan que enfermeros y médicos no están familiarizados con las indicaciones de SVD. Un estudio de cohorte con 388 pacientes mostró que por cada cuatro pacientes, uno se sometió a un sondaje vesical [24,7% (n=96); Intervalo de Confianza del 95% (IC95%): 20% a 29%], y en el 36,5% (IC95%: 33% a 48%) de los casos no presentaban criterios clínicos para dicho procedimiento^(12)^. El uso inadecuado de SVD es más común en servicios de enfermería clínica que de enfermería quirúrgica, y el uso inadecuado del dispositivo se produjo en 14 (10,4%) de los 134 pacientes quirúrgicos, en comparación con 105 (32,4%) de 324 pacientes clínicos, una diferencia del 22% (IC95%: 14,7-29,2; p<0,001)^(13)^.

Respecto al sondaje innecesario, estudios^(14-15)^ con profesionales de la salud con el objetivo de realizar intervenciones para reducir la inserción de SV y, en consecuencia, las complicaciones infecciosas y no infecciosas. Estos implementaron un conjunto de medidas para alinear el conocimiento de médicos y enfermeros sobre las indicaciones de uso de sondas. Se incluyeron situaciones específicas en las que el sondaje urinario es innecesario y acciones educativas que variaron con el tiempo y la metodología. Un conjunto simple de medidas aumentó el uso de SV con indicaciones basadas en evidencia y resultó en un cambio en la práctica relacionada con el uso continuo del dispositivo. Los índices de ITU-AC se mantuvieron en niveles bajos. Sin embargo, no tuvo impacto en las complicaciones no infecciosas^(14-15)^.

Al abordar la preparación del entorno para el procedimiento de inserción de la SVD, se debe realizar una evaluación de la cama del paciente que se someterá al procedimiento. Se recomienda que esta evaluación incluya la cama del paciente (almohada, cojines, sábanas), los suministros médicos hospitalarios disponibles, como, por ejemplo una mesa de apoyo, una fuente de luz y biombos para asegurar la privacidad del paciente.

Respecto a la preparación del paciente para el procedimiento, en cuanto a la higiene del meato urinario, no existe consenso en la literatura sobre la elección de los productos de higiene y las guías difieren en sus recomendaciones. La directriz americana^(3)^ no proporciona una recomendación y afirma que el problema sigue sin solución y presenta una brecha de investigación sobre el uso de soluciones antisépticas. A su vez, la directriz inglesa^(11)^ recomienda el uso de una solución salina para limpiar el meato uretral antes de insertar la sonda.

La HM permeó el subproceso 2. En el entorno hospitalario, la HM es esencial para prevenir daños relacionados con la asistencia a la salud. Requiere un mayor compromiso de todos los involucrados. En ese sentido, las intervenciones que realizan los gestores para asegurar el cumplimiento de la infraestructura y los insumos (lavabos, grifos, dispensadores, toallas de papel) son importantes para asegurar las condiciones necesarias a los profesionales de la salud que prestan atención directa a los pacientes^(16)^.

La seguridad y la calidad del cuidado en el proceso de enfermería relacionado con SVD no solo están relacionadas con la cualificación profesional y con las medidas de prevención. Existen otros factores que pueden incidir en este proceso asistencial, como son los recursos, los materiales suficientes y de calidad, así como una estructura adecuada para llevar a cabo el procedimiento.

Al abordar el procedimiento de inserción, el GT resaltó las competencias del enfermero y los deberes del técnico de enfermería en situaciones en las que se necesita apoyo para realizar el procedimiento de inserción de SVD. En ese sentido, el profesional enfermero debe tener conocimiento científico y habilidad técnica, y debe buscar el equilibrio entre la seguridad del paciente y el costo-beneficio^(17-18)^.

En cuanto a la práctica clínica, la *guideline* de *EuropeanAssociation of Urology Nurses* (EAUN) presenta directrices basadas en evidencia para apoyar una práctica uniforme de inserción de SVD en adultos^(19-20)^. Un estudio sueco evaluó el impacto de las directrices de la EAUN sobre la técnica aséptica durante el sondaje en dos hospitales. El cumplimiento del procedimiento con la técnica aséptica se asoció con los equipos de cirugía y cardiología (*Odds ratio* (OR): 2,35; IC95%: 1,69 - 3,27), al uso de “kit” estéril de sondaje (OR: 2,06; IC95%: 1,42 – 2,97), al uso de campos estériles para la zona de inserción (OR: 1,91; IC95%: 1,24 - 2,96) y al uso del término “técnica estéril” para la inserción de la SV (OR: 1,64; IC95%: 1,11 - 2,43). El resultado demostró que entre el 55% y el 74% de los enfermeros practicaron una o más precauciones que aseguraron una técnica aséptica para la inserción de SV, lo que demuestra una brecha entre las directrices de la EAUN y el desempeño real. Se mencionaron estrategias de mejora relacionadas con el monitoreo de la técnica estéril durante la inserción de la sonda, para permitir la reducción del riesgo de contaminación y para implementar un protocolo de inserción de SV continua con la presencia de dos personas para reducir las ITU-AC^(21).^.

Mientras tanto, no contar con la ayuda del técnico de enfermería durante el procedimiento de inserción de la SVD resalta la necesidad de cooperación durante la ejecución del procedimiento de inserción. Para corroborar este hallazgo, la implementación del protocolo de inserción con dos profesionales indicó que esto, combinado con otras estrategias, como la aplicación de una lista de verificación de tareas, redujo el riesgo de ITU-AC de los pacientes estudiados^(22)^.

En Brasil, la opinión normativa para la actuación del equipo de enfermería en el sondaje vesical establece directrices dirigidas a la seguridad efectiva del paciente sometido al procedimiento^(4)^. Describe que el paso de la SV es exclusivo del enfermero y presenta acciones que deben ser desarrolladas por el equipo de enfermería durante la ejecución del procedimiento.

Sin embargo, se resalta la necesidad de una legislación que establezca la presencia obligatoria de un técnico de enfermería, en lo que respecta a las responsabilidades relacionadas con la preparación del material y del entorno, con la posición del paciente, la apertura de materiales estériles, el destino de los materiales utilizados y de los residuos generados y, cuando sea necesario, el envío al laboratorio del material recogido para su examen al laboratorio. Estas responsabilidades podrán contribuir a reducir la ocurrencia de incidentes y fortalecer la seguridad del paciente, con rigor técnico-científico y trabajo en equipo.

Respecto al mantenimiento de la SVD, la fijación de la sonda es una práctica reconocida como un aspecto importante y esencial para el mantenimiento del dispositivo. Consiste en utilizar un dispositivo para fijar una sonda foley urinaria en la zona interna del mulso (mujer) o en la parte inferior delo abdomen (hombre) del paciente. Una fijación inadecuada puede provocar complicaciones como retirada inadvertida, lesiones en la piel, trauma o erosión uretral^(23)^.

Un estudio canadiense encontró una prevalencia general del 18% (8/44) de fijación de la sonda. De estas, siete se fijaron correctamente, y el principal método de fijación fue un adhesivo comercial (6/8; 75%)^(24)^. La lesión cutánea del dispositivo está relacionada con la presencia prolongada de la sonda o con la falta de fijación de la sonda; esta puede causar molestias, irritación de la piel y del epitelio uretral, eritema en el borde del meato, hasta pérdida total del espesor del tejido, hasta la división completa del pene, la desfiguración del pene y disfunción sexual y urinaria^(25)^.

Las directrices^(3,19,25)^ recomiendan que, si no se puede evitar la colocación de una sonda permanente, esta se fije al cuerpo del paciente para evitar la tracción de la sonda y la presión sobre el meato uretral. La recomendación se basa en un nivel bajo de evidencia. Por lo tanto, se necesitan estudios prospectivos para establecer directrices basadas en evidencias. Los cuidados relacionados con la fijación de la sonda vesical son responsabilidad del equipo de enfermería y deben incluir acciones para prevenir lesiones. En este contexto, concienciar al personal sobre la importancia de las acciones para prevenir las lesiones por presión asociadas al dispositivo invasivo es el primer paso.

Después de lograr su propósito, la SV deberá ser eliminado. El atrapamiento de la SV puede ocurrir debido a un canal de inflado defectuoso, un mal funcionamiento o a la cristalización del fluido dentro del manguito. Las causas de sondas foley retiradas y el método para tratar cada incidente pueden variar

Al recorrer el proceso de SV continuo, es posible percibir una dicotomía entre la práctica, la enseñanza y las evidencias científicas. En ese sentido, se constata que el abordaje de aspectos relacionados con la innovación y la tecnología en las publicaciones asociadas a la gestión de riesgos en el proceso de SV es escaso, al igual que ocurre en otros procedimientos de enfermería realizados de forma rutinaria^(27)^.

Por el contrario, se han investigado acciones para mejorar la práctica clínica y reducir la incidencia de eventos adversos. Estas acciones contemplan principalmente la prevención de factores de riesgos modificables para ITU-AC y trauma uretral^(7,28-30)^. En la literatura se han descrito programas de mejora de la calidad para reducir el uso de SVD mediante conjuntos de intervenciones basados en evidencias, centrados en evitar el uso innecesario, así como en promover la inserción, la técnica y el mantenimiento adecuados de la sonda^(31-32).^.

Las situaciones y razones de omisión del cuidado, que es cuando “el profesional es incapaz de realizar la acción correcta, ya sea en la etapa de planificación, ya sea en la etapa de ejecución”^(33)^, permearon el análisis de riesgos del proceso de SVD. Estos hallazgos están en línea con la literatura que destaca las razones más frecuentes de omisión de los cuidados de enfermería relacionadas con recursos humanos (escasez de fuerza de trabajo), recursos materiales y aumento inesperado de pacientes^(34-35).^.

En ese sentido, el GT reportó la normalización de la desviación, término utilizado para designar un fenómeno que se produce cuando las personas de una organización se vuelven tan insensibles que la práctica desviante ya no les parece incorrecta. La insensibilidad ocurre a diario y, a menudo, se perpetúa en los equipos a lo largo de los años, sin que se le dé la debida importancia hasta que factores críticos se alinean hacia el incidente/daño^(36)^. Ante lo anterior, no preocuparse por la higiene del meato urinario (conocer la técnica y no realizarla) es un ejemplo de desviación de la práctica basada en la evidencia y de violación del control de infección.

Cabe resaltar que los profesionales de la salud no toman decisiones con la intención de aumentar los riesgos asistenciales y causar daño a los pacientes. Sin embargo, la desviación ocurre debido a la ruptura de barreras del proceso o impulsores como el tiempo, el costo, la sobrecarga de trabajo y la presión de los pares^(36)^. En este contexto, evaluar los hallazgos de esta investigación desde la perspectiva de estos conceptos puede ayudar a minimizar o evitar la normalización de la desviación y de la omisión del cuidado. Esto puede lograrse mediante acciones establecidas con transparencia y herramientas de mejora del desempeño, de modo que el cambio y la prevención de las desviaciones comiencen en la línea de frente, con el respaldo de la gestión.

Respecto a la falta de adhesión a las buenas prácticas, es importante observar y estudiar los escenarios de asistencia, los procesos y las relaciones interpersonales para comprender las razones, considerando la complejidad del proceso decisorio y las diferentes percepciones entre los profesionales de la salud.

El SVD es un problema multiprofesional, con responsabilidades compartidas que varían entre las categorías profesionales. Cuando sea necesaria una intervención para generar mejoras, es importante que las medidas de sensibilización y comunicación de nuevas rutinas y estándares se dirijan a todo el equipo de salud, en especial a médicos y enfermeros.

Se espera que este estudio brinde apoyo para repensar los problemas y fallas relacionados con los procesos asistenciales más allá de la enfermería, con miras a minimizar los riesgos en el cuidado y prevenir incidentes de seguridad para los pacientes. Por lo tanto, el compromiso del liderazgo con el uso de SVD, de forma restringida y racional, debe ser visible más allá del grupo multidisciplinario, acercándose al paciente y al acompañante para abrir camino a la mejora.

Respecto a las limitaciones de este estudio, el alcance del AMFE realizado por el GT se limitó al proceso de SVD en adultos, abarcando los cuatro subprocesos presentados. No se incluyeron en el alcance los subprocesos relacionados con la transición del cuidado, el traslado y transporte de pacientes con SVD, con irrigación continua o lavado vesical. No se abordaron grupos de pacientes con rangos de edad específicos (adultos mayores, embarazadas, recién nacidos, bebés y niños), ni tampoco el compromiso de pacientes y familiares.

Como el GT no estaba familiarizado con la herramienta, enfrentó dificultad para utilizarla. La aplicación del método requirió atención debido a la complejidad de los conceptos y de las cadenas de fallas. Sin embargo, tras el período de incorporación y adaptación, con la reanudación del método, siempre que fue necesario, el FMEA posibilitó una discusión colectiva con amplitud reflexiva sobre la práctica diaria asistencial. Dado que los resultados relacionados con la cadena de fallas son inherentes a un hospital universitario, se sugiere ampliar la aplicación de esta herramienta a otras instituciones.

## Conclusión

El análisis proactivo de riesgos asistenciales realizado en el entorno hospitalario fortaleció la aplicabilidad de la herramienta en un proceso de salud. Una temática que requiere mayor visibilidad para contribuir a una cultura de seguridad en el ámbito nacional.

Permitió al GT revisar el proceso de SVD, aumentar la percepción de los riesgos relacionados con la seguridad del paciente y discutir acciones para hacerlo más seguro y calificado. Se mapearon 4 subprocesos, 12 actividades y 70 tareas que pusieron de manifiesto las correlaciones entre los subprocesos/actividades, profesionales de la salud e información. El análisis proactivo de los riesgos asistenciales en el proceso de SVD en el entorno hospitalario reveló 55 MFP, 92 CFP y 40 EFP.

El subproceso 2 presentó en sus cadenas de fallas los mayores riesgos que necesitan ser gestionados de forma proactiva. Se resaltó la posición estratégica de los enfermeros en la toma de decisiones para la gestión de riesgos relacionados con la práctica asistencial del proceso de SV, desde la inserción y el uso continuo hasta la retirada del dispositivo.

Las principales recomendaciones sugeridas por el GT consideraron los modos de falla potenciales con alta PA y formaron una lista para brindar resultados mejorados relacionados con el objeto del estudio. Incluyó acciones relacionadas con la implementación de discusiones multidisciplinarias para evitar el uso innecesario de SVD; revisión del protocolo de prevención de ITU-AC y del POE de inserción de SVD; institucionalización de una lista de verificación de inserción y aplicación de un conjunto de acciones para el mantenimiento y manejo de SVD, acompañado de un programa de capacitación con la participación de la academia. Finalmente, recomendó realizar un estudio de las necesidades de mejoras relacionadas con la infraestructura hospitalaria, la calificación del proceso de adquisición de insumos médicos hospitalarios y el dimensionamiento de los equipos de enfermería.
